# Epidemiology and outcomes of septic shock in Japan: a nationwide retrospective cohort study from a medical claims database by the Japan Sepsis Alliance (JaSA) study group

**DOI:** 10.1186/s13054-025-05556-8

**Published:** 2025-07-16

**Authors:** Taro Imaeda, Takehiko Oami, Tatsuro Yokoyama, Satoshi Nakagawa, Hiroshi Ogura, Nobuaki Shime, Yutaka Umemura, Asako Matsushima, Kiyohide Fushimi, Taka-aki Nakada

**Affiliations:** 1 Department of Emergency and Critical Care Medicine, Chiba University Graduate School of Medicine, 1-8-1 Inohana, Chuo, 260-8677 Chiba, Japan; 2 Iwaki City Medical Center, Critical Care Medicine, Fukushima, Japan; 3 Department of Traumatology and Acute Critical Medicine, Osaka University Graduate School of Medicine, Suita, Osaka, Japan; 4 Department of Emergency and Critical Care Medicine, Graduate School of Biomedical & Health Sciences, Hiroshima University, Hiroshima, Japan; 5 Department of Emergency and Critical Care, Graduate School of Medical Sciences, Nagoya City University, Nagoya, Japan; 6 Institute of Science Tokyo, Department of Health Policy and Informatics, Tokyo, Japan

**Keywords:** Sepsis, Septic shock, In-hospital mortality, Intensive care unit, Japan, Administrative database, Epidemiology

## Abstract

**Background:**

Septic shock is a critical condition associated with high mortality and resource utilization. Although improvements in early recognition and management of sepsis have been reported globally, the trends in clinical outcomes among patients with septic shock in Japan remain unclear.

**Methods:**

We conducted a retrospective cohort study using data from the Japanese Diagnosis Procedure Combination (DPC) nationwide medical claims database from 2010 to 2020. Adult inpatients (aged ≥ 18 years) with sepsis were identified based on the criteria for presumed serious infection (blood culture plus ≥ 4 days of intravenous antibiotics) and acute organ dysfunction, determined using the International Classification of Diseases, 10th Revision codes and treatment procedures. This approach is broadly consistent with the Sepsis-3 definition but adapted for administrative data. Septic shock was defined as the administration of at least one vasopressor, without requiring hypotension or lactate criteria, in line with previous administrative database studies. The primary outcome was in-hospital mortality rate. Secondary outcomes included deaths per 1,000 inpatients, hospital length of stay (LOS), intensive care unit (ICU) admission rates, and ICU LOS. Outcomes among patients with non-shock sepsis were also analyzed for comparison. Trends and subgroup analyses were performed according to age and sex and combinations of ICU admission and shock status.

**Results:**

Among 4,426,342 patients with sepsis, 649,082 (14.7%) had septic shock. In-hospital mortality was significantly higher in the shock group than in the non-shock group (36.5% vs. 20.0%, *P* < 0.001), with a longer median LOS (38 vs. 25 days) and higher ICU admission (50.7% vs. 19.2%). From 2010 to 2020, in-hospital mortality decreased from 46.7 to 33.2% in the shock group and from 26.0 to 18.0% in the non-shock group. The number of deaths per 1,000 inpatients only slightly decreased in the shock group (2.8 to 2.4) but increased in the non-shock group (7.0 to 8.2). The proportion of patients with septic shock increased from 0.64 to 0.83%. Meanwhile, the mean LOS decreased from 61.0 to 53.6 days in the shock group and from 45.7 to 34.4 days in the non-shock group. Only about half of the patients with septic shock were admitted to ICUs, and mortality was higher in non-ICU patients throughout most of the study period, although the difference diminished in 2019. Subgroup analyses showed persistently high mortality rates among older patients (≥ 85 years) and males across the study period.

**Conclusions:**

Despite improvements in survival and LOS over the past decade, septic shock remains a highly lethal and resource-intensive condition in Japan, with in-hospital mortality rates exceeding 30%. Mortality was particularly high among older patients, with rates consistently exceeding 40% among those aged ≥ 85 years.

**Supplementary Information:**

The online version contains supplementary material available at 10.1186/s13054-025-05556-8.

## Background

Sepsis is a life-threatening condition resulting from a dysregulated host response to infection, leading to organ dysfunction and high mortality rates [1]. It remains a major global health burden, with an estimated 48.9 million cases and 11 million sepsis-related deaths annually [2]. Despite advances in early recognition and standardized management protocols, sepsis continues to be the leading cause of death and prolonged hospitalization [3]. The increasing incidence of sepsis has been attributed to an aging population, growing prevalence of chronic diseases, and improved diagnostic sensitivity [4]. Among its clinical manifestations, septic shock represents the most severe and resource-intensive form, characterized by profound circulatory failure and often requiring vasopressors and intensive care.

However, trends in sepsis-related mortality and healthcare utilization vary considerably across healthcare systems and patient populations [5]. Therefore, country-specific data are essential to understand local epidemiology and inform clinical and policy decisions. In Japan, the Diagnostic Procedure Combination (DPC) system, which was introduced in 2003 as a case-mix-based reimbursement and administrative database under the country’s universal health insurance system, provides a robust platform for large-scale epidemiological research. The DPC database includes claims-based data on diagnoses, procedures, examinations, and treatments, enabling a comprehensive analysis of healthcare utilization and clinical outcomes. Previous studies have demonstrated its utility in evaluating infectious disease trends, critical care practices, and patient prognosis [6–8].

Although overall sepsis mortality appears to decline with greater adherence to evidence-based care, the extent to which these improvements have benefited subgroups, such as patients with septic shock, remains unclear [9]. Septic shock, given its extremely high mortality and intensive resource demands, warrants dedicated epidemiological investigation. Understanding how trends in mortality, hospital length of stay (LOS), and healthcare resource use differ between septic shock and non-shock sepsis is essential to enhance the quality of clinical decision-making and develop healthcare policies [10].

Japan, as the world’s most rapidly aging society [11], provides a unique opportunity to investigate the impact of population aging on the epidemiology and outcomes of septic shock. While regional heterogeneity necessitates local data, Japan’s experience may also provide valuable insights for countries that will soon face similar demographic changes. This study aimed to evaluate the national trends in clinical outcomes associated with septic shock in Japan. We tested the hypothesis that in-hospital mortality and deaths per 1,000 inpatients due to septic shock have remained high in Japan over the past decade despite advances in sepsis care. Using a nationwide claims-based database that included more than 80 million inpatient admissions from 2010 to 2020, we analyzed the time trends in these outcomes. The primary outcome of this study was in-hospital mortality in patients with septic shock.

## Materials and methods

### Study design and data source

In this retrospective cohort study, we used data from the DPC database, a comprehensive nationwide medical claims system covering acute care hospitals in Japan. The DPC system, introduced in 2003, serves as a standardized medical reimbursement framework that integrates both clinical and administrative data [12]. As of 2024, the DPC database included 1,786 acute care hospitals and approximately 480,000 beds, covering approximately 85% of all inpatient beds designated for acute care in Japan [13]. This dataset provides detailed patient information, including demographics, hospital admissions and discharges, diagnoses, treatments, procedures, and drug prescriptions, making it a valuable resource for epidemiological research.

### Study population

We included adult patients (aged ≥ 18 years) who were admitted to DPC-participating hospitals between January 1, 2010, and December 31, 2020. We excluded patients who were younger than 18 years or had missing demographic data, such as age, sex, or in-hospital mortality status. To avoid duplicate counting, patients with multiple hospitalizations were excluded from the analysis of in-hospital mortality and deaths per 1,000 inpatients. However, for analysis with regards to the length of hospital stay and proportion of patients with septic shock, each admission was treated as a separate unit of analysis. Patients with sepsis were identified based on established criteria [6]; detailed definitions are provided in the following subsection.

### Definitions of sepsis and septic shock

Patients with sepsis were identified using our previously employed method [6], which combines presumed serious infection and acute organ dysfunction and is broadly aligned with the third International Consensus Definitions for Sepsis and Septic Shock (Sepsis.3) [1]. As defined in a previous study [6], a presumed serious infection was defined based on the presence of a blood culture test and initiation of new intravenous antibiotics for at least four consecutive days. The first antibiotic dose was administered within ± 2 calendar days of blood culture collection. In cases where a patient died before completing four days of antibiotic therapy, the patient was included regardless of treatment duration.

Owing to the absence of laboratory values, such as creatinine, bilirubin, and platelet counts, in the DPC dataset, acute organ dysfunction was identified based on the International Statistical Classification of Diseases and Related Health Problems, 10th Revision (ICD-10) diagnosis codes and organ-specific treatment data. Hepatic dysfunction, thrombocytopenia, coagulopathy, and acidosis were identified using ICD-10 codes (Supplementary Table S3 in Additional File 1), whereas circulatory, respiratory, and renal dysfunctions were assessed based on the initiation of vasopressors, mechanical ventilation or oxygen therapy, and renal replacement therapy, respectively. Patients undergoing maintenance dialysis for end-stage renal disease were excluded from the renal replacement therapy category to ensure the accurate identification of acute renal dysfunction requiring temporary renal support.

Community-onset sepsis was defined as sepsis occurring within 48 h of hospital admission, with both blood culture collection and antibiotic initiation occurring within this timeframe [14].

Among these patients with sepsis, septic shock was defined as the administration of at least one vasopressor. This definition is consistent with prior epidemiological studies using administrative data, where vasopressor use serves as a pragmatic surrogate for circulatory failure in the absence of hemodynamic or lactate information [15, 16]. Given the diversity of clinical practices across institutions and the limitations inherent to claims data, this operational definition was adopted to enable the large-scale identification of patients likely to have experienced septic shock.

### Study variables

The primary outcome of this study was in-hospital mortality in patients with septic shock. Secondary outcomes included deaths due to sepsis per 1,000 inpatients, hospital LOS, intensive care unit (ICU) admission rates, and ICU LOS, all of which were evaluated among patients with septic shock. For contextual comparison, corresponding data from patients with non-shock sepsis were also analyzed. In addition to the outcome measures, baseline characteristics, such as age, sex, and comorbidities, were compared between the two groups.

### Statistical analysis

Descriptive statistics were used to summarize patient characteristics. Continuous variables were presented as mean (standard deviation, SD) or median (interquartile range, IQR), while categorical variables were reported as absolute counts and percentages. Group comparisons between the septic shock and non-shock sepsis cohorts were conducted using chi-square tests for categorical variables and either Student’s *t*-tests or Mann–Whitney *U* tests for continuous variables, depending on data distribution.

Temporal trends from 2010 to 2020 were assessed for all primary and secondary outcomes, including in-hospital mortality, deaths due to sepsis per 1,000 inpatients, and hospital LOS. To explore epidemiological trends in sepsis over time, we also evaluated the annual changes in the proportion of inpatients with sepsis. Linear regression was applied to continuous variables, such as LOS and deaths due to sepsis per 1,000 inpatients, whereas the Cochran–Armitage trend test was used for categorical variables, including in-hospital mortality and the proportion of patients with sepsis among inpatients.

Subgroup analyses were performed using clinically relevant age categories (18–64, 65–74, 75–84, and ≥ 85 years), 10-year age intervals, sex, and combinations of ICU admission and shock status to explore differences in mortality and LOS. In addition, among patients with septic shock, we compared baseline clinical characteristics between those who were admitted to the ICU and those who were not. Univariate analyses were conducted to evaluate differences in age, comorbidities, infection site, and treatment modalities (e.g., mechanical ventilation, renal replacement therapy, and corticosteroid use). Because changes in mortality over time may influence the observed LOS—as deceased patients tend to have shorter LOS, and lower mortality may inflate the mean LOS—we stratified LOS by survival status. ICU and hospital LOS were analyzed separately for survivors and non-survivors in both the septic shock and non-shock sepsis groups, and annual trends within each subgroup were evaluated using linear regression. To further investigate whether year of admission remained significantly associated with in-hospital mortality after adjusting for potential confounding factors, we performed multivariable logistic regression analyses stratified by shock status. Covariates included year of admission, age, sex, body mass index (BMI), community-onset sepsis, the hospital day of antibiotic initiation, comorbidities, infection site, and treatment modalities (mechanical ventilation, renal replacement therapy, hydrocortisone use, and ICU admission). Adjusted odds ratio (OR) and 95% confidence interval (CI) were reported. In addition, to explore potential factors contributing to temporal trends in outcomes, annual changes in demographic characteristics, comorbidities, infection sites, and treatment modalities were evaluated using linear regression for continuous variables and the Cochran–Armitage trend test for categorical variables.

As the data were derived from a comprehensive nationwide claims database, no missing values were present, and no imputation was required. Variables, such as LOS and ICU stay, were reported as both mean  (SD) and median (IQR) to facilitate comparison with previous studies. Variables with non-normal distributions, including patient age and timing of blood culture collection relative to hospital admission, were expressed as median (IQR).

Statistical significance was set at a two-tailed p-value < 0.05. Data processing and statistical analyses were performed using MariaDB v11.7.2 (MariaDB Corporation Ab, Helsinki, Finland) for SQL-based data extraction, pandas v2.2.3 (pandas development team, USA) in Python v3.12.5 (Python Software Foundation, Wilmington, DE, USA) for data manipulation, and GraphPad Prism 10 (GraphPad Software Inc., San Diego, CA, USA) for statistical testing and visualization.

### Ethical considerations

The Institutional Review Board of the Chiba University Graduate School of Medicine approved this study (approval number: 3429). Because the data were anonymized, the requirement for obtaining informed consent from individual patients was waived. This study adhered to the Declaration of Helsinki and relevant ethical guidelines for medical research.

## Results

### Study population

During the study period from 2010 to 2020, 82,170,094 inpatient admissions were screened, of which 4,426,342 (5.4%) were identified as having sepsis. Of these patients, 649,082 (14.7%) were classified into the septic shock group and 3,777,260 (85.3%) were classified into the non-shock sepsis group (Table 1).

**Table 1:** Comparison of clinical characteristics between the shock and non-shock groups

	Whole population	Shock group	Non-shock group	*P* value
Screened total inpatients, n	82,170,094			
Extracted sepsis, n	4,426,342	649,082 (14.7)	3,777,260 (85.3)	< 0.001
Age, yr ^a^	78 (68–85)	75 (65–82)	78 (68–85)	< 0.001
Female, n (%)	1,850,155 (41.8)	246,406 (38.0)	1,603,749 (42.5)	< 0.001
BMI, kg/m² ^a^	21.3 (18.5–24.3)	21.7 (18.9–24.7)	21.2 (18.4–24.3)	< 0.001
Community-onset sepsis, n (%)	2,669,220 (60.3)	281,567 (43.4)	2,387,653 (63.2)	< 0.001
Comorbidity				
Hypertension, n (%)	1,308,553 (29.6)	174,571 (26.9)	1,133,982 (30.0)	< 0.001
Diabetes mellitus, n (%)	870,479 (19.7)	129,809 (20.0)	740,670 (19.6)	< 0.001
Malignant tumor, n (%)	863,783 (19.5)	120,701 (18.6)	743,082 (19.7)	< 0.001
Ischemic heart disease, n (%)	504,441 (11.4)	112,171 (17.3)	392,270 (10.4)	< 0.001
Heart failure, n (%)	383,853 (8.7)	69,204 (10.7)	314,649 (8.3)	< 0.001
Chronic respiratory disease, n (%)	322,246 (7.3)	35,820 (5.5)	286,426 (7.6)	< 0.001
Cerebrovascular disease, n (%)	311,375 (7.0)	51,542 (7.9)	259,833 (6.9)	< 0.001
Chronic renal failure, n (%)	223,415 (5.1)	45,724 (7.0)	177,691 (4.7)	< 0.001
Focus of infection (*n* = 1,361,887)				
Respiratory, n (%)	677,869 (49.8)	41,416 (26.2)	636,453 (52.9)	< 0.001
Abdominal, n (%)	328,037 (24.1)	64,173 (40.7)	263,864 (21.9)	< 0.001
Urogenital, n (%)	102,951 (7.6)	4,834 (3.1)	98,117 (8.2)	< 0.001
Bone and soft tissue, n (%)	73,942 (5.4)	9,442 (6.0)	64,500 (5.4)	< 0.001
Blood, n (%)	1,534 (0.1)	224 (0.1)	1,310 (0.1)	< 0.001
Others, n (%)	177,554 (13.0)	37,747 (23.9)	139,807 (11.6)	< 0.001
The hospital day of the blood culture draw, day ^a^	1 (1–10)	7 (1–22)	1 (1–9)	< 0.001
The hospital day of antibiotic initiation, day ^a^	1 (1–9)	4 (1–17)	1 (1–8)	< 0.001
Length of antibiotic treatment, days				< 0.001
Mean (SD)	15.6 (16.0)	21.7 (23.4)	14.9 (14.2)	
Median (IQR)	11 (7–19)	15 (8–27)	11 (7–17)	
Length of vasopressor use, days				
Mean (SD)	–	4.8 (10.62)	–	–
Median (IQR)	–	2 (1–4)	–	–
Hydrocortisone use, n (%)	295,803 (6.7)	85,563 (13.2)	210,240 (5.6)	< 0.001
Length of hydrocortisone treatment				< 0.001
Mean (SD)	5.2 (9.3)	5.2 (10.2)	5.1 (8.9)	
Median (IQR)	2 (1–5)	3 (1–5)	2 (1–5)	

BMI, body mass index; IQR, interquartile range; SD, standard deviation

^a^ The data shown represent the median value along with the interquartile range

The median age of patients with sepsis was 78 years (IQR: 68–85 years), and the septic shock group was significantly younger (75 years, IQR: 65–82 years) than the non-shock group (78 years, IQR: 68–85 years, *P* < 0.001). The proportion of female patients was significantly lower in the septic shock group (38.0% vs. 42.5%, *P* < 0.001). The prevalence of hypertension, diabetes mellitus, malignant tumors, ischemic heart disease, and heart failure differed significantly between the groups (all *P* < 0.001). Notably, ischemic heart disease (17.3% vs. 10.4%) and chronic renal failure (7.0% vs. 4.7%) were more common in the septic shock group, whereas chronic respiratory disease (5.5% vs. 7.6%) was more frequent in the non-shock group (*P* < 0.001).

The distribution of infection sites also showed significant differences between the groups. Respiratory infections were the most common type of infection in both groups but were significantly lower in the septic shock group (26.2% vs. 52.9%, *P* < 0.001). In contrast, abdominal infections were more common in the septic shock group (40.7% vs. 21.9%, *P* < 0.001).

### Overall In-Hospital mortality, length of stay, and ICU utilization (2010–2020)

Between 2010 and 2020, the in-hospital mortality rates were 36.5% and 20.0% in the septic shock and the non-shock groups, respectively (*P* < 0.001). The number of deaths per 1,000 inpatients was 2.6 in the shock group and 7.6 in the non-shock group (*P* < 0.001). The median LOS was 38 days (IQR, 19–69 days) in the shock group and 25 days (IQR, 14–45 days) in the non-shock group (*P* < 0.001). ICU admission occurred in 50.7% of patients with shock and 19.2% of those without shock (*P* < 0.001). Among patients with septic shock, ICU admission was significantly less frequent in those with malignancy (41.7%) compared to those without malignancy (54.2%) (*P* < 0.001). The median ICU stay was 7 days in the shock group and 4 days in the non-shock group (*P* < 0.001) (Table 2). Among patients with septic shock, those admitted to the ICU were younger, had a lower proportion of females, and were less likely to have community-onset sepsis. They also had higher rates of heart failure, chronic respiratory disease, and chronic renal disease. In terms of infection sites, they more frequently had infections classified as “other,” including central nervous system infections and infective endocarditis. Additionally, the rates of mechanical ventilation, renal replacement therapy, and hydrocortisone use were higher in the shock group (Supplementary Table S4 in Additional file 1).

**Table 2:** Comparison of clinical outcomes between shock and non-shock groups

	Whole population	Shock group	Non-shock group	*P* value
**Primary outcome**				
In-hospital mortality, n (%) ^a^	833,815 (22.6)	212,591 (36.5)	621,224 (20.0)	< 0.001
**Secondary outcomes**				
Deaths per 1,000 inpatients	10.15	2.59	7.56	< 0.001
Length of hospital stay, days				< 0.001
Mean (SD)	41.1 (107.0)	58.0 (130.2)	38.2 (102.2)	
Median (IQR)	26 (14–49)	38 (19–69)	25 (14–45)	
ICU admission, n (%)	1,053,792 (23.81)	329,005 (50.7)	724,787 (19.2)	< 0.001
Length of ICU stay, days				< 0.001
Mean (SD)	6.0 (4.9)	7.8 (5.5)	5.2 (4.5)	
Median (IQR)	4 (2–10)	7 (3–14)	4 (2–8)	

ICU, intensive care unit; IQR, interquartile range; SD, standard deviation

^a^ After excluding repeat hospitalizations, the total number of patients with sepsis was 3,696,098

### Temporal trends in mortality, incidence of sepsis, and length of stay

**Fig. 1:**
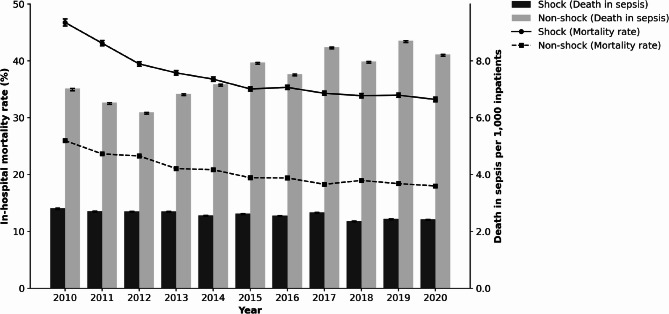
Annual changes in mortality and deaths per 1,000 inpatients in shock and non-shock sepsis This figure presents annual data from 2010 to 2020. Lines represent in-hospital mortality rates (%), showing a statistically significant decreasing trend in both the shock (*Z* = − 49.37, *P* < 0.001) and the non-shock groups (*Z* = − 83.51, *P* < 0.001) based on the Cochran–Armitage trend test. Bars represent deaths per 1,000 inpatients. The rate for patients with shock showed a slight but significant annual decrease (slope = − 0.038, *R*² = 0.77, *P* = 0.0004), whereas that for patients with non-shock sepsis significantly increased over time (slope = + 0.216, *R*² = 0.75, *P* = 0.0005), as determined using linear regression analysis. Error bars indicate 95% confidence intervals

The in-hospital mortality rate for patients with septic shock significantly declined from 46.7% in 2010 to 33.2% in 2020 (*Z* = − 49.37, *P* < 0.001), whereas the mortality rate for patients with non-shock sepsis also showed a significant decreasing trend, from 26.0% in 2010 to 18.0% in 2020 (*Z* = − 83.51, *P* < 0.001). Although the in-hospital mortality rate for patients with septic shock substantially declined, the number of deaths per 1,000 inpatients due to septic shock showed only a modest decrease, from 2.8 to 2.4 over the same period (slope = − 0.038, *R*² = 0.77, *P* = 0.0004). In contrast, for non-shock sepsis, although the in-hospital mortality rate declined, the number of deaths per 1,000 inpatients increased from 7.0 to 8.2 (slope = + 0.216, *R*² = 0.75, *P* = 0.0005) (Fig. 1). To determine whether the observed improvement in mortality was attributable to changes in patient background or treatment practices, we conducted multivariable logistic regression analysis. After adjusting for age, sex, BMI, community-onset sepsis, the hospital day of antibiotic initiation, comorbidities, infection site, and treatment modalities (mechanical ventilation, renal replacement therapy, hydrocortisone use, and ICU admission), the year of admission remained an independent predictor of in-hospital mortality. The adjusted OR per year were 0.972 (95% CI: 0.971–0.973, *P* < 0.001) in the whole population, 0.960 (95% CI: 0.958–0.962, *P* < 0.001) in the shock group, and 0.977 (95% CI: 0.976–0.978, *P* < 0.001) in the non-shock group (Supplementary Table S5 in Additional file 1).

**Fig. 2:**
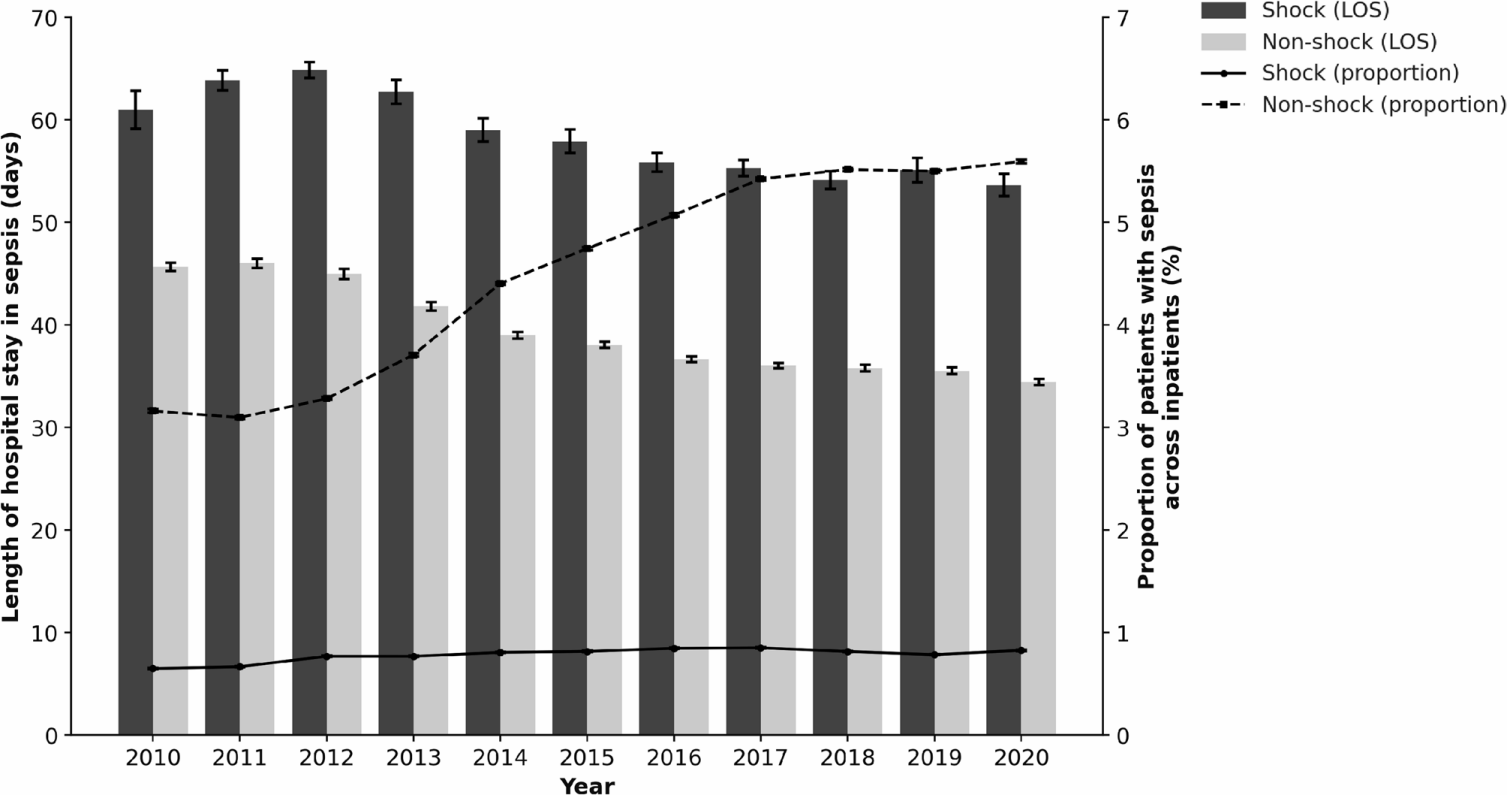
Annual changes in hospital stay and sepsis proportion in shock and non-shock sepsis This figure presents annual data from 2010 to 2020. The lines represent the proportion of patients with sepsis among all inpatients (%), which significantly increased in both the shock (*Z* = 42.95, *P* < 0.0001) and the non-shock groups (*Z* = 387.79, *P* < 0.0001), based on the Cochran–Armitage trend test. Bars represent the mean hospital length of stay (LOS, days), which significantly decreased in both the shock (slope = − 1.11 days/year, *R²* = 0.82, *P* = 0.0001) and the non-shock groups (slope = − 1.27 days/year, *R²* = 0.92, *P* < 0.0001), as determined using linear regression analysis. Error bars indicate 95% confidence intervals

Over the study period, the proportion of patients with septic shock among all inpatients increased significantly from 0.64% in 2010 to 0.83% in 2020 (*Z* = 42.95, *P* < 0.0001). The proportion of patients with non-shock sepsis also significantly increased from 3.16 to 5.59% (*Z* = 387.79, *P* < 0.0001). However, the mean LOS for patients with septic shock decreased from 61.0 days in 2010 to 53.6 days in 2020 (slope = − 1.11 days/year, *R*² = 0.82, *P* = 0.0001), whereas for patients with non-shock sepsis, the decrease was more pronounced, from 45.7 days to 34.4 days (slope = − 1.27 days/year, *R*² = 0.92, *P* < 0.0001) (Fig. 2). Because changes in mortality over time may influence the observed hospital LOS—as deceased patients tend to have shorter stays, and declining mortality may inflate the mean LOS—we analyzed temporal trends in hospital LOS separately for survivors and non-survivors. In the shock group, hospital LOS significantly declined in both survivors and non-survivors (slope = − 1.25 and − 0.87 days/year, respectively; *P* < 0.0001 for both), while in the non-shock group, hospital LOS also significantly declined in both subgroups (slope = − 1.27 and − 0.94 days/year, respectively; *P* < 0.0001 for both) (Supplementary Table S6 in Additional File 1). To explore factors potentially contributing to the temporal improvements in mortality, we evaluated annual trends in patient characteristics and treatment practices. Between 2010 and 2020, the proportion of patients with community-onset sepsis markedly increased (from 34.4 to 49.9% in the shock group), and the timing of antibiotic initiation became earlier (median hospital day: from 7.0 to 3.0). The use of renal replacement therapy and hydrocortisone increased, while mechanical ventilation use remained stable. The distribution of infection sites shifted, with a decline in respiratory infections and increases in abdominal and urogenital infections. Baseline demographics also changed over time, with slight increases in age and BMI, and a higher prevalence of chronic renal failure (Supplementary Table S7 in Additional file 1).

### Subgroup analyses according to age and sex, and ICU admission status

**Fig. 3:**
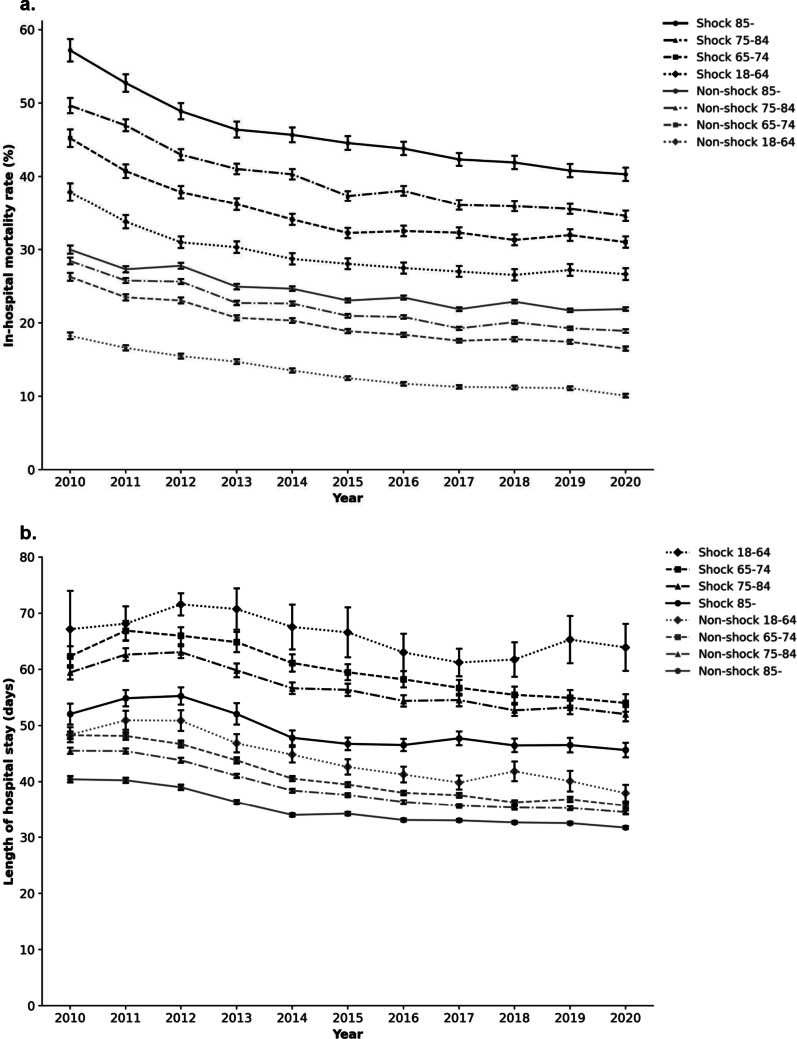
**A**. Annual changes in in-hospital mortality by age among patients with septic shock and non-shock sepsis. This figure presents annual data from 2010 to 2020. Patients were stratified into four age groups: 18–64, 65–74, 75–84, and ≥ 85 years. Across all age categories, older patients consistently exhibited higher in-hospital mortality rates in both groups. A significant decreasing trend was observed in all age subgroups (*Z* = − 20.05 to − 58.27, *P* < 0.001). Black lines represent patients with septic shock, and gray lines represent those with non-shock sepsis. Line styles and marker shapes indicate age subgroups: solid lines with circles represent ≥ 85 years, dash-dot lines with triangles represent 75–84 years, dashed lines with squares represent 65–74 years, and dotted lines with diamonds represent 18–64 years. Error bars indicate 95% confidence intervals. Statistical trends for each subgroup: Shock group 18–64: *Z* = − 20.05, *P* < 0.0001 65–74: *Z* = − 26.31, *P* < 0.0001 75–84: *Z* = − 34.83, *P* < 0.0001 ≥ 85: *Z* = − 25.28, *P* < 0.0001 Non-shock group 18–64: *Z* = − 48.37, *P* < 0.0001 65–74: *Z* = − 47.33, *P* < 0.0001 75–84: *Z* = − 58.27, *P* < 0.0001 ≥ 85: *Z* = − 41.60, *P* < 0.0001. **B**. Annual changes in hospital stay by age among patients with septic shock and non-shock sepsis This figure presents annual data from 2010 to 2020. Patients were stratified into four age groups: 18–64, 65–74, 75–84, and ≥ 85 years. The mean hospital length of stay (LOS, days) significantly declined across all age subgroups in both the shock and the non-shock groups. In patients with septic shock, LOS decreased by − 0.73 to − 1.27 days/year depending on the age group. In patients with non-shock sepsis, the decline ranged from − 0.91 to − 1.40 days/year. Black lines represent patients with septic shock, and gray lines represent those with non-shock sepsis. Line styles and marker shapes indicate age subgroups: solid lines with circles represent ≥ 85 years, dash-dot lines with triangles represent 75–84 years, dashed lines with squares represent 65–74 years, and dotted lines with diamonds represent 18–64 years. Error bars indicate 95% confidence intervals Shock group 18–64: slope = − 0.73, *R*² = 0.51, *P* = 0.0014 65–74: slope = − 1.27, *R*² = 0.85, *P* < 0.0001 75–84: slope = − 1.08, *R*² = 0.84, *P* < 0.0001 ≥ 85: slope = − 0.93, *R*² = 0.73, *P* < 0.0001 Non-shock group 18–64: slope = − 1.27, *R*² = 0.87, *P* < 0.0001 65–74: slope = − 1.40, *R*² = 0.92, *P* < 0.0001 75–84: slope = − 1.22, *R*² = 0.91, *P* < 0.0001 ≥ 85: slope = − 0.91, *R*² = 0.88, *P* < 0.0001

Older age was consistently associated with higher in-hospital mortality in both septic shock and non-shock sepsis groups, as shown by age-stratified annual mortality trends (Fig. 3a). In all four age categories (18–64, 65–74, 75–84, and ≥ 85 years), the in-hospital mortality rate showed a significant decreasing trend over time (*Z* = − 20.05 to − 58.27, *P* < 0.001). Among patients with septic shock aged ≥ 85 years, although a significant downward trend was observed (*Z* = − 25.28, *P* < 0.001), the mortality rate remained persistently high, exceeding 40% throughout the study period (Supplementary Figure S2a in Additional file 3). In contrast, the mortality rate for patients with non-shock sepsis aged ≥ 85 years declined from 30.0% in 2010 to 21.9% in 2020, reflecting a statistically significant improvement in survival (*Z* = − 41.60, *P* < 0.001). Corresponding trends were also observed in hospital LOS. In both septic shock and non-shock sepsis groups, the mean hospital LOS declined consistently across all age categories (Fig. 3b).

## Discussion

We analyzed the nationwide trends in the epidemiology and outcomes of patients with septic shock in Japan between 2010 and 2020. Although septic shock consistently accounts for approximately 15% of all sepsis cases, its absolute incidence has increased, as sepsis has become more common. In-hospital mortality declined from 46.7 to 33.2% but remained high despite advances in care. As expected, patients with septic shock had longer hospital and ICU stays and higher ICU admission rates, reflecting the intensive resource demands of this condition. Outcomes varied according to age and sex, highlighting the ongoing clinical and systemic burden of septic shock and the need for focused strategies to improve care.

In our study, 14.7% of the patients with sepsis were classified as having septic shock. This proportion is lower than that reported in ICU-based cohorts in Japan, such as the JSEPTIC DIC study (2011–2013, 33.9%) [17] and the FORECAST study (2016–2017, 62.9%) [18], both of which focused on critically ill patients admitted to ICUs and likely reflected populations that were more severely affected. In addition, these studies did not use the Sepsis-3 definition, and their findings are therefore not directly comparable to ours. Notably, our study is among the first large-scale epidemiological investigations in Japan to use the nationwide DPC administrative claims database, capturing over 4.4 million patients with sepsis across all levels of hospital care. Therefore, this study offers valuable insights into the real-world burden of septic shock in a broader inpatient population. International comparisons show considerable variation: in England (2011–2015), 19.9% of Sepsis-3-defined patients with sepsis had septic shock [19]; in France (2010–2015), 66.8% were classified as such based on ICD-coded data [20]; and in the United States (US) (2016–2017), 20% of patients with sepsis progressed to shock during hospitalization [21]. In Germany, a 2019 emergency department study reported a 24.0% prevalence of Sepsis-3-defined sepsis cases [22]. These differences reflect the varying case definitions, clinical settings, and data sources.

In our study, the in-hospital mortality rate in patients with septic shock was 36.5%, showing a substantial decline from 46.7% in 2010 to 33.2% in 2020. Previous studies have reported a global downward trend in mortality attributed to earlier recognition and advances in critical care [6]. However, when focusing specifically on septic shock, the mortality rates vary considerably depending on the region and definition. A retrospective observational study conducted at a tertiary emergency center in the US between 2010 and 2015 reported a 28-day mortality rate of 23.4% in patients with septic shock defined according to the Sepsis-3 criteria [23]. Similarly, in Asia, a meta-analysis from Korea reported an in-hospital mortality rate of 34.3 % [24], and nationwide health insurance data from Taiwan showed a decline in in-hospital mortality from 27.2% in 2002 to 21.1% in 2012 [25], suggesting improvements in management. By contrast, in England, while Sepsis-2-defined mortality decreased from 37 to 33% between 2011 and 2015, mortality among Sepsis-3-defined cases remained consistently high at approximately 56% [19]. Furthermore, nationwide data from France showed a case fatality rate of 44–46% for septic shock between 2010 and 2015, with only limited improvement observed [20].

Although in-hospital mortality rates declined, the reduction in deaths per 1,000 inpatients due to septic shock was statistically significant but modest in magnitude, decreasing from 2.8 in 2010 to 2.4 in 2020. This finding suggests that the increasing number of sepsis cases offset the increase in individual survival. In contrast, deaths per 1,000 inpatients due to non-shock sepsis have increased significantly from 7.0 in 2010 to 9.4 in 2020, highlighting the growing burden of less severe presentations. These findings were consistent with those of previous studies. In the US, sepsis-related deaths increased by approximately 1.3-fold from 2010 to 2019 [26]. Similarly, in Spain, sepsis-related deaths per 1,000 inpatients rose from 4.0 to 5.7 between 2005 and 2019, with the absolute number doubling during this period [27]. These trends are in contrast with the Global Burden of Disease estimates, indicating a worldwide decline in sepsis mortality [2]. Variations in case definitions and population coverage are likely to account for these discrepancies.

In our study, the year of admission remained a significant independent predictor of lower in-hospital mortality, even after adjusting for key covariates (adjusted OR per year: 0.960 [95% CI: 0.958–0.962] in the shock group). This finding suggests that the observed reduction in mortality was not solely due to changes in patient demographics or baseline characteristics but may reflect improvements in clinical care practices over the decade. Several temporal changes support this hypothesis. From 2010 to 2020, the proportion of patients with community-onset sepsis markedly increased (from 34.4 to 49.9% in the shock group), and the timing of antibiotic initiation became earlier (median hospital day: from 7.0 to 3.0), indicating improvements in early recognition and treatment. In addition, the use of organ support therapies evolved; for example, the proportion of shock patients receiving mechanical ventilation remained stable while the use of renal replacement therapy and hydrocortisone increased. There were also shifts in infection site distributions, with a decline in respiratory infections and an increase in abdominal and urogenital infections, possibly reflecting improved infection control and coding accuracy. Baseline characteristics also changed over time: the median age increased slightly (from 74 to 76 years in the shock group), and BMI increased marginally, while the prevalence of certain comorbidities, such as chronic renal failure, also rose. However, despite an aging population and increasing comorbidity burden, the mortality declined—suggesting that improvements in sepsis recognition, antibiotic stewardship, and supportive care may have played a critical role. These trends emphasize the importance of system-wide improvements in sepsis care, beyond changes in case mix. In particular, the progressive adoption of evidence-based sepsis guidelines, including the Surviving Sepsis Campaign, may have contributed to earlier diagnosis, timely antibiotic administration, and standardized supportive care [28]. Although recent evaluations have indicated suboptimal adherence to such bundles in real-world clinical settings [29], the widespread dissemination of sepsis protocols and associated education efforts may still have driven incremental improvements in overall care quality over time.

Our study showed that patients with septic shock had substantially higher ICU admission rates (50.7%) than those without shock (19.2%). However, this rate is still markedly lower than that reported in the US, where up to 55–65% of patients with septic shock are managed in ICUs [30, 31]. The relatively low ICU admission rate in Japan may reflect structural constraints, including a limited number of ICU beds (5 beds per 100,000 population in Japan vs. 30 in the US) [32, 33], as well as cultural and institutional preferences for non-ICU management of certain populations, such as older individuals or those with malignancies [34]. In line with this, we found that only 41.7% of patients with septic shock and cancer were admitted to the ICU compared with 54.2% of patients without cancer. Consequently, nearly half of the patients with septic shock were managed outside of the ICU, reflecting the broader use of general wards and intermediate care units for these patients in Japan. Furthermore, among patients with septic shock, the cumulative in-hospital mortality from 2010 to 2020 was significantly higher in those admitted to the ICU (34.9%) compared to those not admitted (38.2%) (*P* < 0.001). When examining temporal trends, ICU admission was significantly associated with lower in-hospital mortality between 2010 and 2018. However, this difference diminished in recent years and was no longer statistically significant in 2019 and 2020. This trend may reflect evolving ICU triage practices and improved care delivery in non-ICU settings, particularly in the context of resource constraints and advances in early recognition and ward-based sepsis management [35, 36]. These observations may offer valuable implications for countries with limited ICU bed availability, such as Japan, suggesting that improved outcomes may be achieved through strategies that enhance the quality of non-ICU sepsis care.

In this study, the median LOS for patients with septic shock was 38 days, with a mean LOS decreasing from 61.0 days in 2010 to 53.6 days in 2020. Despite this improvement, the LOS in Japan remains substantially longer than that in Western countries. For example, a French national study (2010–2015) reported a median LOS of 20 days [20], and German administrative data (2007–2013) showed a median LOS of approximately 21 days [37]. In the US, a 2016 study using the NEDS database reported a median LOS of 10–16 days [38]. These differences likely reflect variations in healthcare systems, ICU capacities, discharge practices, and cultural attitudes. Japan’s average hospital LOS is among the highest in countries belonging to the Organisation for Economic Co-operation and Development, with 16.1 days compared to 7.5 in Germany, 5.6 in France, and 6.0 in the US [39]. Longer stays may be attributed to the integrated nature of acute and rehabilitative care and the widespread use of long-term care beds. Conditions such as septic shock result in significantly prolonged hospitalization.

In the present study, differences in patient outcomes were observed according to age and sex. Although all age groups showed significant decreases in in-hospital mortality over time, the mortality rate among patients aged ≥ 75 years with septic shock remained high throughout the study period. Notably, in 2020, the final year of the study, the in-hospital mortality rate for this age group still exceeded 36%, despite the overall declining trend. These findings indicate that although survival has generally improved, older patients with septic shock continue to face a disproportionately high risk of death. This aligns with previous findings suggesting a limited benefit of sepsis guideline implementation in the oldest age group [40, 41]. In contrast, younger patients (e.g., those aged 20–39 years) showed greater relative improvements in survival, possibly reflecting differences in treatment responsiveness or physiological reserves. Sex-based subgroup analyses revealed that in-hospital mortality rates were consistently higher in male patients than in female patients in both the septic shock and the non-shock groups. Although the mortality rates significantly declined over time in both sexes, this sex difference persisted throughout the study period. In 2020, the mortality rate among male patients with septic shock was 34.4% compared to 31.4% in females. These findings are consistent with previous evidence indicating sex-related differences in immune responses, comorbidity burden, and healthcare utilization patterns [42]. Although our study was descriptive, these observations suggest that patient characteristics, such as age and sex, may influence clinical outcomes, highlighting the importance of personalized approaches for sepsis management in older populations.

This study had some limitations. First, the DPC database did not include laboratory data, such as serum creatinine, bilirubin, platelet counts, or lactate levels. Because Sepsis-3 definitions require laboratory values to assess organ dysfunction, we were unable to apply the full Sepsis-3 criteria. Instead, we defined sepsis based on a combination of diagnostic codes and treatment data related to organ support, following the methods used in previous epidemiological studies [5]. This approach may have underestimated the incidence of sepsis compared to definitions using laboratory data. In contrast, a recent report [43] found that the Rhee criteria [5], which define suspected infection using a combination of culture and antibiotic orders, have significantly lower sensitivity but higher specificity than approaches using electronic health record data. Given the similarity of our approach, we expect our cohort to represent a more specific and severely ill population. Second, the definition of septic shock was based solely on the administration of vasopressors, without accompanying data on hypotension or serum lactate levels, which may have led to misclassification. Third, the database lacks standardized severity scores such as the Sequential Organ Failure Assessment score or the Acute Physiology and Chronic Health Evaluation II score, limiting our ability to directly assess illness severity. However, among patients with septic shock, those who were not admitted to the ICU had lower rates of organ support interventions—such as mechanical ventilation and renal replacement therapy—and a shorter duration of vasopressor use. These findings suggest that the non-ICU group may have been less severely ill, although this interpretation should be made with caution due to the absence of objective severity measures. Fourth, the DPC database does not contain information on treatment limitation policies (e.g., do-not-resuscitate orders or end-of-life care decisions), which may have influenced ICU admission and management strategies, particularly in older patients or those with advanced comorbidities. Fifth, the study was retrospective in design and was based on administrative claims data, which may have involved misclassification, coding variability, and lack of clinical granularity. Sixth, although the DPC dataset is the largest nationwide inpatient database in Japan and includes approximately 85% of all inpatient beds designated for acute care, it may not fully represent sepsis care in long-term care facilities or non-participating institutions. Seventh, the median duration of antibiotic treatment was 11 days (15 days in the shock group), which was longer than that reported in recent randomized controlled trials [44]. This may be partly due to the inclusion of older patients and those with respiratory infections, which often require prolonged treatment courses [45, 46]. Eighth, blood culture results were not available, and bloodstream infection was defined using diagnostic codes, which may have underestimated its incidence. Finally, subgroup analyses of mortality according to age and sex, and ICU admission combined with shock status were based on crude in-hospital mortality rates without adjustment for patient severity or treatment differences owing to limitations in the available data.

## Conclusions

Based on nationwide data from 2010 to 2020, septic shock in Japan remains a critical condition with a high mortality rate (36.5%), which is significantly higher than that of non-shock sepsis. Despite modest improvements over the past decade, targeted strategies and system-level efforts are essential to reduce this burden and improve outcomes. In-hospital mortality increased notably with age, particularly among patients with septic shock. These findings emphasize the importance of age-specific strategies and may inform healthcare planning in other rapidly aging countries like Japan.

## Electronic supplementary material

Additional file 1: Table S1.Comorbidity categories and corresponding ICD-10 codes. Table S2. Focus on infections with the corresponding ICD-10 codes. Table S3. Acute organ dysfunction categories with corresponding ICD-10 codes. Table S4. Comparison of clinical characteristics between ICU and non-ICU patients among those with septic shock. Table S5.Multivariable logistic regression analysis for in-hospital mortality in patients with sepsis, stratified by shock status, adjusting for demographics, comorbidities, infection characteristics, and treatment modalities. Table S6. Length of ICU and hospital stay among patients with and without septic shock. a. ICU length of stay by group and year. b. Hospital length of stay by group and year. Table S7. Patient characteristics, comorbidities, infection sites, and treatments among patients with sepsis in Japan (2010–2020). a. Temporal trends. b. Trend analysis.

Additional file 2: Figure S1. Annual changes in sepsis cases and deaths in shock and non-shock sepsis. This figure presents annual data from 2010 to 2020. The number of patients with sepsis significantly increased in both the shock group (slope = +4,180.55 patients/year, *R*² = 0.69, *P*= 0.0018) and the non-shock group (slope = +24,439.73 patients/year, *R*²= 0.81, *P* = 0.0002). The number of deaths significantly increased in the non-shock group (slope =+3,224.08 deaths/year, *R*² = 0.73, *P* = 0.0008), while the trend in the shock group remained relatively stable (slope = +329.01 deaths/year, *R*² = 0.16, *P* = 0.2288). Marker shapes indicate the data type: circles denote the number of patients, and triangles indicate the number of deaths. Gray lines indicate patients with the non-shock group, and black lines indicate patients in the shock group. Error bars indicate 95% confidence intervals.

Additional file 3: Figure S2a.Annual trends in in-hospital mortality by age group in shock and non-shock sepsis. This figure presents annual data from 2010 to 2020. In-hospital mortality rates are shown by age group stratified in 10-year intervals (≤29, 30–39, 40–49, 50–59, 60–69, 70–79, and ≥80 years) among patients with septic shock and non-shock sepsis. Among patients with septic shock, all age groups, except for those aged ≤29 years, showed a significant decreasing trend in in-hospital mortality, with the strongest trend seen in patients aged ≥80 years (*Z* =–34.848, *P* < 0.0001). In non-shock patients with sepsis, all age groups exhibited significant downward trends (*P* < 0.0001 for all). Error bars indicate 95% confidence intervals. Statistical trends for each subgroup: Shock group≤29: *Z*=–1.897, *P* = 0.0578 30–39: *Z*=–3.614, *P* = 0.0003 40–49: *Z*=–6.882, *P* < 0.0001 50–59: *Z*=–12.827, *P* < 0.0001 60–69: *Z*=–20.937, *P* < 0.0001 70–79: *Z*=–30.125, *P* < 0.0001≥80: *Z*=–34.848, *P* < 0.0001 Non-shock group≤29: *Z*=–8.217, *P* < 0.0001 30–39: *Z*=–10.584, *P* < 0.0001 40–49: *Z*=–16.426, *P* < 0.0001 50–59: *Z*=–29.384, *P* < 0.0001 60–69: *Z*=–42.594, *P* < 0.0001 70–79: *Z*=–50.512, *P* < 0.0001≥80: *Z*=–54.085, *P* < 0.0001 Figure S2b.Annual changes in hospital stay by age group in shock and non-shock sepsis. This figure presents annual data from 2010 to 2020. The mean length of hospital stay (LOS, days) is shown by age group stratified in 10-year intervals (≤29, 30–39, 40–49, 50–59, 60–69, 70–79, and ≥80 years) among patients with septic shock and non-shock sepsis. In the shock group, a significant decrease in LOS was observed in patients aged 50–59 years and older, whereas there were no significant trends among younger age groups (≤49 years). In the non-shock group, LOS significantly decreased over time in all age groups, except for those aged ≤29 years (*P* = 0.3465). The steepest decline in LOS was observed in patients aged 30–39 years (slope =–1.99 days/year, *R*²= 0.57). Error bars indicate 95% confidence intervals. Statistical trends for each subgroup: Shock group≤29: slope =–0.66, *R*² = 0.01, *P* = 0.7362 30–39: slope =–0.10, *R*² = 0.01, *P* = 0.9120 40–49: slope =–0.26, *R*² = 0.04, *P* = 0.5457 50–59: slope =–1.28, *R*² = 0.65, *P* = 0.0026 60–69: slope =–0.94, *R*² = 0.67, *P* = 0.0021 70–79: slope =–1.19, *R*² = 0.80, *P* = 0.0002≥80: slope =–1.06, *R*² = 0.86, *P* < 0.0001 Non-shock group≤29: slope = 0.61, *R*² = 0.10, *P* = 0.3465 30–39: slope =–1.99, *R*² = 0.57, *P* = 0.0074 40–49: slope =–1.18, *R*² = 0.77, *P* = 0.0004 50–59: slope =–1.43, *R*² = 0.88, *P* < 0.0001 60–69: slope =–1.34, *R*² = 0.94, *P* < 0.0001 70–79: slope =–1.37, *R*² = 0.92, *P* < 0.0001≥80: slope =–1.03, *R*² = 0.89, *P* < 0.0001

Additional file 4: Figure S3a. Annual changes in in-hospital mortality by sex in shock and non-shock sepsis. This figure presents annual data from 2010 to 2020. In-hospital mortality rates are shown by sex among patients with septic shock and non-shock sepsis. Male patients exhibited consistently higher in-hospital mortality rates than female patients in both groups throughout the study period. Although the mortality rates significantly declined in all subgroups, male patients in the septic shock group showed the highest mortality rate (from 47.7% in 2010 to 34.4% in 2020), followed by female patients in the same group (from 45.2% to 31.4%). Similar decreasing trends were observed in patients with non-shock sepsis (male: from 28.0% to 19.7%; female: from 23.2% to 15.8%). Black lines represent patients with septic shock, and gray lines represent those with non-shock sepsis. Male patients are denoted by solid lines with circles, and female patients by dashed lines with squares. Error bars indicate 95% confidence intervals. Figure S3b.Annual changes in hospital stay by sex in shock and non-shock sepsis. This figure presents annual data from 2010 to 2020.Mean hospital length of stay (LOS, days) is shown by sex among patients with septic shock and non-shock sepsis. While the LOS was longer in male patients than in female patients in both groups, all subgroups demonstrated significant annual reductions. LOS decreased from 60.7 to 52.8 days in male patients with septic shock (slope =–1.14 days/year, *R*² = 0.80, *P* = 0.0002), from 61.5 to 55.0 days in female patients with septic shock (slope =–1.06 days/year, *R*² = 0.83, *P*= 0.0001), from 45.8 to 34.3 days in male patients with non-shock sepsis (slope =–1.28 days/year, *R*² = 0.92, *P* < 0.0001), and from 45.5 to 34.5 days in female patients with non-shock sepsis (slope =–1.25 days/year, *R*² = 0.90, *P* < 0.0001). Black lines represent patients with septic shock, and gray lines represent those with non-shock sepsis. Male patients are represented by solid lines with circles, and female patients by dashed lines with squares. Error bars indicate 95% confidence intervals

Additional file 5: Figure S4.Annual changes in in-hospital mortality by ICU admission and shock status. This figure presents annual data from 2010 to 2020. In-hospital mortality rates are shown across four subgroups based on ICU admission and shock status: shock with ICU admission, shock without ICU admission, non-shock with ICU admission, and non-shock without ICU admission. Throughout most of the study period, patients with septic shock who were not admitted to the ICU exhibited the highest mortality rates (from 50.5% in 2010 to 33.1% in 2020), whereas non-shock patients admitted to the ICU consistently showed the lowest mortality (from 22.9% to 17.1%). The gap in mortality between ICU and non-ICU subgroups within the shock group narrowed over time, with no statistically significant difference observed in 2019 and 2020. Black lines represent patients with septic shock, and gray lines represent those with non-shock sepsis. ICU-admitted patients are denoted by solid lines with circles, and non-ICU patients by dashed lines with squares. Error bars indicate 95% confidence intervals

## Data Availability

No datasets were generated or analysed during the current study.

## Abbreviations

BMI: Body Mass Index
DPC: Diagnosis Procedure Combination
ICD-10: International Statistical Classification of Diseases and Related Health Problems, 10th Revision
ICU: Intensive Care Unit
IQR: Interquartile Range
LOS: Length Of Stay
SD: Standard Deviation
Sepsis-3: Third International Consensus Definitions for Sepsis and Septic Shock
US: United States
