# Supplementary material for: Epidemiology and outcomes of septic shock in Japan: a nationwide retrospective cohort study from a medical claims database by the Japan Sepsis Alliance (JaSA) study group
Source: Crit Care. 2025 Jul 16;29:309. doi: 10.1186/s13054-025-05556-8 (PMC12269265; doi:10.1186/s13054-025-05556-8)
Supplement: Supplementary file 1 — Additional file 1: Table S1.Comorbidity categories and corresponding ICD-10 codes. Table S2. Focus on infections with the corresponding ICD-10 codes. Table S3. Acute organ dysfunction categories with corresponding ICD-10 codes. Table S4. Comparison of clinical characteristics between ICU and non-ICU patients among those with septic shock. Table S5.Multivariable logistic regression analysis for in-hospital mortality in patients with sepsis, stratified by shock status, adjusting for demographics, comorbidities, infection characteristics, and treatment modalities. Table S6. Length of ICU and hospital stay among patients with and without septic shock. a. ICU length of stay by group and year. b. Hospital length of stay by group and year. Table S7. Patient characteristics, comorbidities, infection sites, and treatments among patients with sepsis in Japan (2010–2020). a. Temporal trends. b. Trend analysis. [file 13054_2025_5556_MOESM1_ESM.docx]

**Epidemiology and outcomes of septic shock in Japan: a nationwide retrospective cohort study from a medical claims database by the Japan Sepsis Alliance (JaSA) study group**

Taro Imaeda, Takehiko Oami, Tatsuro Yokoyama, Satoshi Nakagawa, Hiroshi Ogura, Nobuaki Shime, Yutaka Umemura, Asako Matsushima, Kiyohide Fushimi, Taka-aki Nakada

***Online data Supplement***

**Table S1.** Comorbidity categories and corresponding ICD-10 codes

| Comorbidity | ICD-10 codes |
| --- | --- |
| Malignant tumor | C00-C97, D00-D09 |
| Hypertension | I10-I15 |
| Diabetes mellitus | E10-E14 |
| Heart failure | I50 |
| Cerebrovascular disease | I60-I69 |
| Ischemic heart disease | I20-I25 |
| Chronic respiratory disease | J40-J47 |
| Chronic renal failure | N18 |

**Table S2.** Focus of infections with the corresponding ICD-10 codes

| Focus of infection | ICD-10 codes |
| --- | --- |
| Respiratory | A15-A16, J00-J06, J09-J18, J20-J22, J31-J32, J35-J37, J39.0, J39.1, J85-J86 |
| Urogenital | A18.1, A51.0, A54.0-A54.2, A56.0-A56.2, A59.0, A60.0, N30.0, N30.8, N39.0, N41.0-N41.3, N45, N49.0-N49.2, N70-N77, O23 |
| Abdominal | A00-A09, A18.3, A42.1, A74.8, K35-K38, K57.0, K57.2, K57.4, K57.8, K61, K63.0, K63.1, K65, K67, K75.0, K80.0, K80.1, K80.3, K80.4, K81, K83.0 |
| Bone and soft tissue | A18.0, A18.4, A26.0, A28.1, A31.1, A31.8, A32.0, A36.3, A42.2, A43.1, A46, A48.0, L00-L08, M00, M01.0, M46.3, M46.5, M49.1-M49.3, M60.0, M86.0, M86.1, M86.65, M86.66, M86.69, M86.99 |
| Blood | A19 (Miliary tuberculosis), A40.0 (Invasive group A streptococcal disease), A49.0 (Methicillin susceptible Staphylococcus aureus bacteremia), A49.1 (Invasive pneumococcal disease), A49.9 (bacteremia) |

**Table S3.** Acute organ dysfunction categories with corresponding ICD-10 codes

| Organ dysfunction | ICD-10 codes |
| --- | --- |
| Renal | N00.9 (Acute nephritis syndrome) |
|  | N10 (Acute tubulointerstitial nephritis) |
|  | N17.0 (Shock kidney, Acute parenchymal renal failure, Acute kidney tubular necrosis) |
|  | N17.1 (Acute renal cortical necrosis) |
|  | N17.8 (Acute prerenal failure) |
|  | N17.9 (Acute kidney injury) |
| Hepatic | K72.0 (Acute liver failure) |
|  | K72.9 (Liver failure, details unknown) |
|  | K76.8 (Shock liver) |
| Thrombocytopenia | D69.5 (Secondary thrombocytopenia) |
|  | D69.6 (Thrombocytopenia) |
| Coagulopathy | D65 (Disseminated intravascular coagulation) |
|  | D68.9 (Other and unspecified coagulation disorders) |
| Acidosis | E87.2 (Acidosis, metabolic or lactic) |

**Table S4.** Comparison of clinical characteristics between ICU and non-ICU patients among those with septic shock

|  | **Shock group** | **ICU group** | **Non-ICU group** | ***P* value** |
| --- | --- | --- | --- | --- |
| Extracted sepsis, n | 649,082 | 329,005 | 320,077 | < 0.001 |
| Age, yr ^a^ | 75 (65–82) | 73 (63–80) | 77 (68–84) | < 0.001 |
| Female, n (%) | 246,406 (38.0) | 117,449 (35.7) | 128,957 (40.3) | < 0.001 |
| BMI, kg/m² ^a^ | 21.7 (18.9–24.7) | 22.0 (19.3–25.0) | 21.3 (18.6–24.2) | < 0.001 |
| Community-onset sepsis, n (%) | 281,567 (43.4) | 138,360 (42.1) | 143,207 (44.7) | < 0.001 |
| Comorbidity |  |  |  |  |
| Hypertension, n (%) | 174,571 (26.9) | 92,205 (28.0) | 82,366 (25.7) | < 0.001 |
| Diabetes mellitus, n (%) | 129,809 (20.0) | 63,487 (19.3) | 66,322 (20.7) | < 0.001 |
| Malignant tumor, n (%) | 120,701 (18.6) | 44,344 (13.5) | 76,357 (23.9) | < 0.001 |
| Ischemic heart disease, n (%) | 112,171 (17.3) | 72,907 (22.2) | 39,264 (12.3) | < 0.001 |
| Heart failure, n (%) | 69,204 (10.7) | 42,077 (12.8) | 27,127 (8.5) | < 0.001 |
| Chronic respiratory disease, n (%) | 35,820 (5.5) | 20,076 (6.1) | 15,744 (4.9) | < 0.001 |
| Cerebrovascular disease, n (%) | 51,542 (7.9) | 30,876 (9.4) | 20,666 (6.5) | < 0.001 |
| Chronic renal failure, n (%) | 45,724 (7.0) | 25,629 (7.8) | 20,095 (6.3) | < 0.001 |
| Focus of infection (n = 157,836) |  |  |  |  |
| Respiratory, n (%) | 41,416 (26.2) | 15,815 (24.2) | 25,601 (27.7) | < 0.001 |
| Abdominal, n (%) | 64,173 (40.7) | 22,148 (33.9) | 42,025 (45.4) | < 0.001 |
| Urogenital, n (%) | 4,834 (3.1) | 1,296 (2.0) | 3,538 (3.8) | < 0.001 |
| Bone and soft tissue, n (%) | 9,442 (6.0) | 3,420 (5.2) | 6,022 (6.5) | < 0.001 |
| Blood, n (%) | 224 (0.1) | 98 (0.2) | 126 (0.1) | 0.50 |
| Others, n (%) | 37,747 (23.9) | 22,492 (34.5) | 15,255 (16.5) | < 0.001 |
| The hospital day of the blood culture draw, day ^a^ | 7 (1–22) | 8 (1-21) | 6 (1-23) | < 0.001 |
| The hospital day of antibiotic initiation, day ^a^ | 4 (1–17) | 5 (1-16) | 4 (1-19) | < 0.001 |
| Length of antibiotic treatment, days |  |  |  | < 0.001 |
| Mean (SD) | 21.7 (23.4) | 22.9 (24.4) | 20.5 (22.3) |  |
| Median (IQR) | 15 (8–27) | 16 (9–29) | 14 (8–25) |  |
| Length of vasopressor use, days |  |  |  | < 0.001 |
| Mean (SD) | 4.8 (10.6) | 5.2 (10.4) | 4.4 (10.9) | – |
| Median (IQR) | 2 (1–4) | 2 (1-5) | 1 (1-4) | – |
| Mechanical ventilation, n (%) | 327,080 (50.4) | 243,156 (73.9) | 83,924 (26.2) | < 0.001 |
| Duration of mechanical ventilation, days |  |  |  | < 0.001 |
| Mean (SD) | 17.8 (49.2) | 16.4 (35.1) | 21.8 (76.3) |  |
| Median (IQR) | 7 (3-17) | 7 (3–17) | 7 (2–18) |  |
| Renal replacement therapy, n (%) | 118,801 (18.3) | 90,258 (27.4) | 28,543 (8.9) | < 0.001 |
| Duration of renal replacement therapy, days |  |  |  | < 0.001 |
| Mean (SD) | 11.2 (14.8) | 11.2 (13.8) | 11.4 (17.5) |  |
| Median (IQR) | 7 (3-14) | 7 (3–14) | 7 (3–14) |  |
| Hydrocortisone use, n (%) | 85,563 (13.2) | 59,084 (18.0) | 26,479 (8.3) | < 0.001 |
| Length of hydrocortisone treatment |  |  |  | 0.59 |
| Mean (SD) | 5.2 (10.2) | 5.0 (9.6) | 5.8 (11.3) |  |
| Median (IQR) | 3 (1–5) | 3 (1-5) | 2 (1-6) |  |

BMI, body mass index; ICU: intensive care unit; IQR, interquartile range; SD, standard deviation

^a^ The data shown represent the median value along with the interquartile range.

**Table S5.** Multivariable logistic regression analysis for in-hospital mortality in patients with sepsis, stratified by shock status, adjusting for demographics, comorbidities, infection characteristics, and treatment modalities

| **Variable** | **OR (95% CI)** | | |
| --- | --- | --- | --- |
|  | **Whole population** | **Shock group** | **Non-shock group** |
| Year | 0.972 (0.971–0.973) | 0.960 (0.958–0.962) | 0.977 (0.976–0.978) |
| Age | 1.034 (1.034–1.035) | 1.027 (1.027–1.028) | 1.038 (1.037–1.038) |
| Sex (male) | 1.234 (1.227–1.241) | 1.121 (1.107–1.135) | 1.267 (1.259–1.275) |
| BMI | 0.955 (0.954–0.955) | 0.975 (0.974–0.976) | 0.949 (0.948–0.949) |
| Community-onset sepsis | 0.923 (0.917–0.929) | 1.265 (1.248–1.282) | 0.891 (0.884–0.897) |
| Comorbidity |  |  |  |
| Hypertension | 0.528 (0.524–0.531) | 0.590 (0.582–0.598) | 0.522 (0.518–0.526) |
| Diabetes mellitus | 0.941 (0.935–0.948) | 0.935 (0.921–0.950) | 0.949 (0.942–0.957) |
| Malignant tumor | 2.486 (2.469–2.504) | 1.343 (1.322–1.365) | 2.894 (2.871–2.916) |
| Ischemic heart disease | 0.765 (0.758–0.772) | 0.791 (0.778–0.804) | 0.730 (0.722–0.738) |
| Heart failure | 1.137 (1.127–1.148) | 1.038 (1.018–1.058) | 1.155 (1.143–1.168) |
| Chronic respiratory disease | 1.031 (1.020–1.041) | 1.223 (1.193–1.254) | 1.035 (1.024–1.047) |
| Cerebrovascular disease | 1.065 (1.054–1.076) | 1.028 (1.006–1.051)^*^ | 1.085 (1.072–1.098) |
| Chronic renal failure | 1.360 (1.344–1.376) | 1.193 (1.166–1.221) | 1.499 (1.479–1.520) |
| Focus of infection |  |  |  |
| Abdominal (vs. Respiratory) | 0.644 (0.635–0.653) | 0.308 (0.299–0.318) | 0.672 (0.661–0.683) |
| Urogenital (vs. Respiratory) | 0.609 (0.595–0.624) | 0.749 (0.697–0.804) | 0.589 (0.574–0.605) |
| Bone and soft tissue (vs. Respiratory) | 0.633 (0.615–0.651) | 0.450 (0.424–0.478) | 0.606 (0.586–0.627) |
| Blood (vs. Respiratory) | 3.446 (3.080–3.856) | 2.779 (2.022–3.819) | 3.572 (3.164–4.032) |
| Others (vs. Respiratory) | 1.441 (1.420–1.463) | 0.980 (0.947–1.013)^**^ | 1.408 (1.383–1.433) |
| The hospital day of antibiotic initiation | 1.006 (1.005–1.006) | 1.004 (1.004–1.004) | 1.006 (1.006–1.006) |
| Mechanical ventilation | 3.977 (3.947–4.008) | 3.257 (3.209–3.305) | 3.503 (3.471–3.536) |
| Renal replacement therapy | 2.281 (2.257–2.304) | 2.547 (2.507–2.588) | 1.783 (1.757–1.808) |
| Hydrocortisone use | 1.450 (1.436–1.463) | 1.596 (1.569–1.623) | 1.277 (1.262–1.292) |
| ICU admission | 0.650 (0.645–0.655) | 0.457 (0.451–0.464) | 0.681 (0.675–0.687) |

BMI, body mass index; CI, confidence interval; ICU, intensive care unit; OR, odds ratio

All variables were statistically significant (*P* < 0.001), except cerebrovascular disease (* *P* = 0.01; statistically significant) and others (vs. respiratory) (** *P* = 0.23; not statistically significant) in the shock group.

**Table S6.** Length of ICU and hospital stay among patients with and without septic shock

1. ICU length of stay by group and year

| **Group** | **Year** | **Survivors (n)** | **Survivors ICU-LOS** | | **Non-survivors (n)** | **Non-survivors ICU-LOS** | |
| --- | --- | --- | --- | --- | --- | --- | --- |
|  |  |  | **mean (SD)** | **median (IQR)** |  | **mean (SD)** | **median (IQR)** |
| Shock | 2010 | 8,096 | 7.6 (5.4) | 7 (3–14) | 5,375 | 8.7 (5.5) | 9 (3–14) |
|  | 2011 | 13,696 | 7.8 (5.4) | 7 (3–14) | 8,269 | 8.6 (5.5) | 9 (3–14) |
|  | 2012 | 18,482 | 7.9 (5.7) | 7 (3–14) | 9,673 | 8.7 (5.7) | 9 (3–14) |
|  | 2013 | 19,008 | 7.9 (5.6) | 7 (3–14) | 9,808 | 8.5 (5.6) | 9 (3–14) |
|  | 2014 | 22,413 | 7.5 (5.3) | 7 (3–13) | 10,967 | 8.4 (5.4) | 8 (3–14) |
|  | 2015 | 23,391 | 7.5 (5.3) | 7 (3–13) | 10,970 | 8.4 (5.7) | 8 (3–14) |
|  | 2016 | 25,145 | 7.5 (5.3) | 7 (3–13) | 11,309 | 8.3 (5.5) | 8 (3–14) |
|  | 2017 | 24,237 | 7.4 (5.3) | 6 (3–13) | 11,221 | 8.3 (5.6) | 8 (3–14) |
|  | 2018 | 23,247 | 7.3 (5.2) | 6 (3–13) | 10,677 | 8.1 (5.5) | 8 (3–14) |
|  | 2019 | 21,333 | 7.5 (5.2) | 7 (3–13) | 10,435 | 8.1 (5.4) | 8 (3–14) |
|  | 2020 | 21,021 | 7.6 (5.5) | 7 (3–13) | 10,232 | 8.5 (6.5) | 8 (3–14) |
| Non-shock | 2010 | 19,578 | 5.5 (4.6) | 4 (2–8) | 5,145 | 6.4 (4.8) | 5 (2–11) |
|  | 2011 | 31,048 | 5.5 (4.6) | 4 (2–8) | 7,657 | 6.4 (5.0) | 5 (2–11) |
|  | 2012 | 39,038 | 5.5 (4.6) | 4 (2–8) | 8,747 | 6.2 (5.0) | 4 (2–11) |
|  | 2013 | 45,940 | 5.3 (4.4) | 4 (2–8) | 9,655 | 6.2 (4.9) | 4 (2–10) |
|  | 2014 | 58,501 | 5.1 (4.4) | 3 (2–7) | 11,748 | 5.9 (4.9) | 4 (2–10) |
|  | 2015 | 64,619 | 5.0 (4.3) | 3 (2–7) | 12,163 | 6.0 (4.8) | 4 (2–10) |
|  | 2016 | 68,483 | 5.0 (4.3) | 3 (2–7) | 12,886 | 5.8 (4.8) | 4 (2–9) |
|  | 2017 | 71,534 | 4.9 (4.3) | 3 (1–7) | 13,565 | 5.7 (4.7) | 4 (2–9) |
|  | 2018 | 71,241 | 4.8 (4.2) | 3 (2–7) | 13,475 | 5.6 (4.7) | 4 (2–9) |
|  | 2019 | 68,208 | 4.9 (4.2) | 3 (2–7) | 13,767 | 5.7 (4.6) | 4 (2–9) |
|  | 2020 | 64,859 | 5.0 (4.5) | 3 (2–7) | 12,930 | 5.9 (5.4) | 4 (2–9) |

ICU: intensive care unit; IQR, interquartile range; LOS, length of stay; SD, standard deviation

Linear trend in ICU length of stay was assessed using linear regression. The estimated annual change (slope), model fit (*R²*), and *P* value were:
– Shock group – Survivors: slope = –0.04 days/year, *R²* = 0.14, *P* = 0.2590
– Shock group – Non-survivors: slope = –0.13 days/year, *R²* = 0.70, *P* = 0.0013
– Non-shock group – Survivors: slope = –0.13 days/year, *R²* = 0.70, *P* = 0.0013
– Non-shock group – Non-survivors: slope = –0.08 days/year, *R²* = 0.45, *P* = 0.0239

1. Hospital length of stay by group and year

| **Group** | **Year** | **Survivors (n)** | **Survivors Hospital-LOS** | | **Non-survivors (n)** | **Non-survivors Hospital-LOS** | |
| --- | --- | --- | --- | --- | --- | --- | --- |
|  |  |  | **mean (SD)** | **median (IQR)** |  | **mean (SD)** | **median (IQR)** |
| Shock | 2010 | 15,769 | 62.8 (58.4) | 48 (28–81) | 12,090 | 58.7 (227.7) | 34 (14–68) |
|  | 2011 | 26,859 | 67.4 (65.8) | 49 (28–84) | 18,423 | 58.7 (146.4) | 33 (13–68) |
|  | 2012 | 35,620 | 67.7 (77.5) | 49 (28–83) | 20,741 | 60.0 (115.6) | 32 (13–70) |
|  | 2013 | 36,152 | 65.2 (109.6) | 46 (26–79) | 19,951 | 58.3 (182.0) | 30 (12–65) |
|  | 2014 | 42,876 | 60.1 (83.7) | 42 (24–73) | 21,890 | 56.9 (219.9) | 28 (11–61) |
|  | 2015 | 44,968 | 58.5 (88.9) | 42 (24–71) | 22,010 | 56.8 (235.2) | 28 (11–59) |
|  | 2016 | 48,636 | 57.4 (109.0) | 41 (23–70) | 23,646 | 52.7 (153.4) | 27 (10–58) |
|  | 2017 | 47,565 | 55.9 (65.1) | 40 (23–68) | 22,736 | 53.9 (159.4) | 26 (10–57) |
|  | 2018 | 45,793 | 55.1 (65.2) | 39 (22–68) | 21,324 | 52.1 (177.4) | 25 (9–55) |
|  | 2019 | 41,141 | 55.2 (75.6) | 39 (22–68) | 19,763 | 54.9 (241.3) | 24 (8–54) |
|  | 2020 | 41,378 | 53.4 (93.3) | 37 (21–65) | 19,751 | 54.1 (196.6) | 23 (9–52) |
| Non-shock | 2010 | 105,538 | 42.9 (44.4) | 29 (16–54) | 30,168 | 55.2 (137.4) | 34 (15–66) |
|  | 2011 | 165,981 | 43.6 (75.9) | 29 (16–53) | 44,333 | 55.2 (169.3) | 32 (14–63) |
|  | 2012 | 192,454 | 42.2 (53.6) | 29 (16–52) | 48,164 | 56.0 (247.2) | 31 (14–60) |
|  | 2013 | 220,208 | 39.7 (65.2) | 27 (15–49) | 50,633 | 50.8 (205.9) | 29 (13–57) |
|  | 2014 | 291,628 | 37.1 (74.8) | 25 (14–45) | 62,119 | 48.0 (164.6) | 27 (12–54) |
|  | 2015 | 322,735 | 36.0 (64.9) | 24 (14–44) | 66,779 | 48.2 (183.8) | 27 (12–52) |
|  | 2016 | 361,802 | 34.7 (62.3) | 24 (14–43) | 71,407 | 46.7 (190.7) | 26 (12–51) |
|  | 2017 | 375,827 | 34.0 (47.6) | 23 (14–42) | 72,583 | 46.3 (194.5) | 25 (11–50) |
|  | 2018 | 380,043 | 33.6 (59.1) | 23 (13–41) | 74,060 | 47.2 (238.5) | 25 (11–48) |
|  | 2019 | 356,153 | 33.3 (72.2) | 23 (13–41) | 70,920 | 46.7 (229.3) | 24 (11–48) |
|  | 2020 | 344,551 | 32.5 (46.2) | 22 (13–39) | 69,174 | 44.1 (221.2) | 22 (10–44) |

IQR, interquartile range; LOS, length of stay; SD, standard deviation

Linear trend in hospital length of stay was assessed using linear regression. The estimated annual change (slope), model fit (*R²*), and *P* value were:
– Shock group – Survivors: slope = –1.25 days/year, *R²* = 0.92, *P* < 0.0001
– Shock group – Non-survivors: slope = –0.87 days/year, *R²* = 0.92, *P* < 0.0001
– Non-shock group – Survivors: slope = –1.27 days/year, *R²* = 0.93, *P* < 0.0001
– Non-shock group – Non-survivors: slope = –0.94 days/year, *R²* = 0.92, *P* < 0.0001

**Table S7.** Patient characteristics, comorbidities, infection sites, and treatments among patients with sepsis in Japan (2010–2020)

1. Temporal trends

| **Category** | **2010** | **2011** | **2012** | **2013** | **2014** | **2015** | **2016** | **2017** | **2018** | **2019** | **2020** |
| --- | --- | --- | --- | --- | --- | --- | --- | --- | --- | --- | --- |
| Age ^a^ |  |  |  |  |  |  |  |  |  |  |  |
| Whole population | 76 (66-83) | 76 (66-84) | 76 (65-83) | 76 (66-84) | 77 (67-84) | 77 (67-85) | 78 (68-85) | 78 (68-85) | 78 (69-86) | 79 (70-86) | 79 (70-86) |
| Shock group | 74 (65-81) | 75 (65-82) | 74 (64-81) | 74 (64-81) | 74 (65-82) | 74 (65-82) | 75 (66-82) | 75 (66-83) | 75 (66-83) | 76 (67-83) | 76 (67-83) |
| Non-shock group | 76 (66-83) | 77 (66-84) | 76 (65-84) | 77 (66-84) | 77 (67-85) | 78 (67-85) | 78 (68-85) | 78 (69-86) | 79 (69-86) | 79 (70-86) | 79 (70-87) |
| Sex (male) |  |  |  |  |  |  |  |  |  |  |  |
| Whole population, n (%) | 97,362 (59.5) | 151,686 (59.3) | 177,259 (59.7) | 193,630 (59.2) | 243,924 (58.3) | 265,863 (58.2) | 292,561 (57.9) | 299,784 (57.8) | 299,784 (57.5) | 279,426 (57.3) | 274,908 (57.9) |
| Shock group, n (%) | 17,433 (62.6) | 28,325 (62.6) | 35,209 (62.5) | 34,963 (62.3) | 40,379 (62.3) | 41,704 (62.3) | 44,601 (61.7) | 43,471 (61.8) | 41,402 (61.7) | 37,499 (61.6) | 37,690 (61.7) |
| Non-shock group, n (%) | 79,929 (58.9) | 123,361 (58.7) | 142,050 (59.0) | 158,667 (58.6) | 203,545 (57.5) | 224,159 (57.5) | 247,960 (57.2) | 256,313 (57.2) | 258,382 (56.9) | 241,927 (56.6) | 237,218 (57.3) |
| BMI ^a^ |  |  |  |  |  |  |  |  |  |  |  |
| Whole population | 21.2 (18.4-24.2) | 21.2 (18.4-24.2) | 21.2 (18.5-24.2) | 21.2 (18.5-24.2) | 21.3 (18.5-24.2) | 21.3 (18.5-24.3) | 21.3 (18.5-24.3) | 21.3 (18.5-24.4) | 21.3 (18.5-24.4) | 21.3 (18.5-24.4) | 21.4 (18.6-24.5) |
| Shock group | 21.4 (18.7-24.4) | 21.5 (18.8-24.5) | 21.6 (18.8-24.5) | 21.6 (18.9-24.6) | 21.6 (18.9-24.6) | 21.7 (19.0-24.6) | 21.6 (18.9-24.5) | 21.7 (18.9-24.7) | 21.7 (19.0-24.7) | 21.8 (19.1-24.8) | 21.9 (19.1-25.0) |
| Non-shock group | 21.1 (18.3-24.1) | 21.1 (18.4-24.1) | 21.1 (18.4-24.1) | 21.1 (18.4-24.2) | 21.2 (18.4-24.2) | 21.2 (18.4-24.2) | 21.2 (18.4-24.2) | 21.2 (18.4-24.3) | 21.2 (18.5-24.3) | 21.3 (18.5-24.4) | 21.3 (18.5-24.5) |
| Community-onset sepsis |  |  |  |  |  |  |  |  |  |  |  |
| Whole population, n (%) | 78,853 (48.2) | 128,666 (50.3) | 150,303 (50.6) | 178,203 (54.5) | 245,336 (58.6) | 274,646 (60.2) | 312,166 (61.8) | 327,633 (63.2) | 336,745 (64.6) | 324,082 (66.4) | 312,587 (65.8) |
| Shock group, n (%) | 9,579 (34.4) | 16,429 (36.3) | 20,544 (36.5) | 22,211 (39.6) | 27,478 (42.4) | 28,895 (43.1) | 31,808 (44.0) | 32,328 (46.0) | 31,665 (47.2) | 30,141 (49.5) | 30,489 (49.9) |
| Non-shock group, n (%) | 69,274 (51.0) | 112,237 (53.4) | 129,759 (53.9) | 155,992 (57.6) | 217,858 (61.6) | 245,751 (63.1) | 280,358 (64.7) | 295,305 (65.9) | 305,080 (67.2) | 293,941 (68.8) | 282,098 (68.2) |
| Comorbidity |  |  |  |  |  |  |  |  |  |  |  |
| Hypertension |  |  |  |  |  |  |  |  |  |  |  |
| Whole population, n (%) | 39,950 (24.4) | 64,234 (25.1) | 74,603 (25.1) | 84,521 (25.9) | 112,438 (26.9) | 121,544 (26.6) | 162,420 (32.1) | 169,193 (32.6) | 168,712 (32.4) | 158,051 (32.4) | 152,887 (32.2) |
| Shock group, n (%) | 6,460 (23.2) | 10,802 (23.9) | 13,325 (23.6) | 13,448 (24.0) | 15,821 (24.4) | 16,425 (24.5) | 21,424 (29.6) | 21,016 (29.9) | 19,880 (29.6) | 17,724 (29.1) | 18,246 (29.8) |
| Non-shock group, n (%) | 33,490 (24.7) | 53,432 (25.4) | 61,278 (25.5) | 71,073 (26.2) | 96,617 (27.3) | 105,119 (27.0) | 140,996 (32.5) | 148,177 (33.0) | 148,832 (32.8) | 140,327 (32.9) | 134,641 (32.5) |
| Diabetes mellitus |  |  |  |  |  |  |  |  |  |  |  |
| Whole population, n (%) | 29,161 (17.8) | 47,958 (18.8) | 53,827 (18.1) | 59,949 (18.3) | 77,430 (18.5) | 86,088 (18.9) | 102,225 (20.2) | 106,617 (20.6) | 107,089 (20.5) | 100,604 (20.6) | 99,531 (21.0) |
| Shock group, n (%) | 4,947 (17.8) | 8,678 (19.2) | 10,535 (18.7) | 10,589 (18.9) | 12,115 (18.7) | 12,803 (19.1) | 15,000 (20.8) | 14,888 (21.2) | 14,197 (21.2) | 12,937 (21.2) | 13,120 (21.5) |
| Non-shock group, n (%) | 24,214 (17.8) | 39,280 (18.7) | 43,292 (18.0) | 49,360 (18.2) | 65,315 (18.5) | 73,285 (18.8) | 87,225 (20.1) | 91,729 (20.5) | 92,892 (20.5) | 87,667 (20.5) | 86,411 (20.9) |
| Malignant tumor |  |  |  |  |  |  |  |  |  |  |  |
| Whole population, n (%) | 38,432 (23.5) | 56,831 (22.2) | 69,452 (23.4) | 72,784 (22.3) | 86,140 (20.6) | 91,060 (19.9) | 95,334 (18.9) | 96,418 (18.6) | 94,662 (18.2) | 82,382 (16.9) | 80,288 (16.9) |
| Shock group, n (%) | 5,885 (21.1) | 9,158 (20.2) | 12,082 (21.4) | 11,697 (20.8) | 12,621 (19.5) | 12,735 (19.0) | 13,358 (18.5) | 12,418 (17.7) | 11,782 (17.6) | 9,629 (15.8) | 9,336 (15.3) |
| Non-shock group, n (%) | 32,547 (24.0) | 47,673 (22.7) | 57,370 (23.8) | 61,087 (22.6) | 73,519 (20.8) | 78,325 (20.1) | 81,976 (18.9) | 84,000 (18.7) | 82,880 (18.3) | 72,753 (17.0) | 70,952 (17.1) |
| Ischemic heart disease |  |  |  |  |  |  |  |  |  |  |  |
| Whole population, n (%) | 17,900 (10.9) | 28,231 (11.0) | 32,432 (10.9) | 35,775 (10.9) | 45,748 (10.9) | 49,387 (10.8) | 61,232 (12.1) | 63,049 (12.2) | 61,113 (11.7) | 55,964 (11.5) | 53,610 (11.3) |
| Shock group, n (%) | 4,676 (16.8) | 7,641 (16.9) | 9,034 (16.0) | 9,374 (16.7) | 10,969 (16.9) | 11,464 (17.1) | 13,124 (18.2) | 12,801 (18.2) | 11,992 (17.9) | 10,621 (17.4) | 10,475 (17.1) |
| Non-shock group, n (%) | 13,224 (9.7) | 20,590 (9.8) | 23,398 (9.7) | 26,401 (9.7) | 34,779 (9.8) | 37,923 (9.7) | 48,108 (11.1) | 50,248 (11.2) | 49,121 (10.8) | 45,343 (10.6) | 43,135 (10.4) |
| Heart failure |  |  |  |  |  |  |  |  |  |  |  |
| Whole population, n (%) | 14,424 (8.8) | 22,596 (8.8) | 23,866 (8.0) | 26,505 (8.1) | 33,999 (8.1) | 36,762 (8.1) | 46,478 (9.2) | 48,029 (9.3) | 46,353 (8.9) | 43,783 (9.0) | 41,058 (8.6) |
| Shock group, n (%) | 2,961 (10.6) | 4,680 (10.3) | 5,643 (10.0) | 5,700 (10.2) | 6,654 (10.3) | 6,878 (10.3) | 8,325 (11.5) | 7,878 (11.2) | 7,483 (11.1) | 6,578 (10.8) | 6,424 (10.5) |
| Non-shock group, n (%) | 11,463 (8.4) | 17,916 (8.5) | 18,223 (7.6) | 20,805 (7.7) | 27,345 (7.7) | 29,884 (7.7) | 38,153 (8.8) | 40,151 (9.0) | 38,870 (8.6) | 37,205 (8.7) | 34,634 (8.4) |
| Chronic respiratory disease |  |  |  |  |  |  |  |  |  |  |  |
| Whole population, n (%) | 4,895 (3.0) | 8,474 (3.3) | 9,970 (3.4) | 12,274 (3.8) | 17,097 (4.1) | 18,971 (4.2) | 22,896 (4.5) | 23,991 (4.6) | 76,257 (14.6) | 70,893 (14.5) | 56,528 (11.9) |
| Shock group, n (%) | 585 (2.1) | 1,064 (2.3) | 1,342 (2.4) | 1,444 (2.6) | 1,685 (2.6) | 1,824 (2.7) | 2,159 (3.0) | 2,196 (3.1) | 8,700 (13.0) | 7,773 (12.8) | 7,048 (11.5) |
| Non-shock group, n (%) | 4,310 (3.2) | 7,410 (3.5) | 8,628 (3.6) | 10,830 (4.0) | 15,412 (4.4) | 17,147 (4.4) | 20,737 (4.8) | 21,795 (4.9) | 67,557 (14.9) | 63,120 (14.8) | 49,480 (12.0) |
| Cerebrovascular disease |  |  |  |  |  |  |  |  |  |  |  |
| Whole population, n (%) | 12,293 (7.5) | 19,418 (7.6) | 21,273 (7.2) | 22,779 (7.0) | 29,028 (6.9) | 30,600 (6.7) | 37,619 (7.4) | 37,265 (7.2) | 35,725 (6.9) | 33,684 (6.9) | 31,691 (6.7) |
| Shock group, n (%) | 2,263 (8.1) | 3,893 (8.6) | 4,504 (8.0) | 4,294 (7.7) | 4,946 (7.6) | 4,927 (7.4) | 6,137 (8.5) | 5,700 (8.1) | 5,310 (7.9) | 4,824 (7.9) | 4,744 (7.8) |
| Non-shock group, n (%) | 10,030 (7.4) | 15,525 (7.4) | 16,769 (7.0) | 18,485 (6.8) | 24,082 (6.8) | 25,673 (6.6) | 31,482 (7.3) | 31,565 (7.0) | 30,415 (6.7) | 28,860 (6.8) | 26,947 (6.5) |
| Chronic renal failure |  |  |  |  |  |  |  |  |  |  |  |
| Whole population, n (%) | 6,183 (3.8) | 9,370 (3.7) | 11,163 (3.8) | 11,587 (3.5) | 14,558 (3.5) | 15,204 (3.3) | 17,808 (3.5) | 17,330 (3.3) | 41,893 (8.0) | 39,445 (8.1) | 38,874 (8.2) |
| Shock group, n (%) | 1,472 (5.3) | 2,468 (5.5) | 3,041 (5.4) | 2,883 (5.1) | 3,227 (5.0) | 3,337 (5.0) | 3,882 (5.4) | 3,474 (4.9) | 7,753 (11.6) | 7,031 (11.5) | 7,156 (11.7) |
| Non-shock group, n (%) | 4,711 (3.5) | 6,902 (3.3) | 8,122 (3.4) | 8,704 (3.2) | 11,331 (3.2) | 11,867 (3.0) | 13,926 (3.2) | 13,856 (3.1) | 34,140 (7.5) | 32,414 (7.6) | 31,718 (7.7) |
| Focus of infection |  |  |  |  |  |  |  |  |  |  |  |
| Respiratory |  |  |  |  |  |  |  |  |  |  |  |
| Whole population, n (%) | 25,377 (53.1) | 40,828 (53.8) | 43,720 (51.4) | 48,916 (51.3) | 65,735 (52.0) | 72,203 (52.0) | 84,183 (52.8) | 86,323 (52.4) | 89,580 (53.4) | 83,134 (52.0) | 59,428 (42.1) |
| Shock group, n (%) | 2,422 (36.8) | 3,887 (35.1) | 4,483 (33.6) | 4,272 (32.5) | 4,868 (31.5) | 4,877 (30.5) | 5,429 (31.6) | 5,266 (30.7) | 5,218 (30.9) | 4,712 (29.8) | 3,951 (25.9) |
| Non-shock group, n (%) | 22,955 (55.7) | 36,941 (57.0) | 39,237 (54.7) | 44,644 (54.4) | 60,867 (54.8) | 67,326 (54.8) | 78,754 (55.4) | 81,057 (55.0) | 84,362 (56.0) | 78,422 (54.5) | 55,477 (44.1) |
| Abdominal |  |  |  |  |  |  |  |  |  |  |  |
| Whole population, n (%) | 10,080 (21.1) | 15,985 (21.1) | 19,509 (22.9) | 22,275 (23.4) | 30,194 (23.9) | 33,518 (24.1) | 37,708 (23.7) | 39,656 (24.1) | 39,337 (23.5) | 38,757 (24.3) | 41,018 (29.1) |
| Shock group, n (%) | 1,986 (30.2) | 3,511 (31.7) | 4,596 (34.5) | 4,857 (36.9) | 6,292 (40.7) | 6,761 (42.3) | 7,181 (41.8) | 7,394 (43.1) | 7,167 (42.5) | 7,072 (44.8) | 7,356 (48.2) |
| Non-shock group, n (%) | 8,094 (19.7) | 12,474 (19.2) | 14,913 (20.8) | 17,418 (21.2) | 23,902 (21.5) | 26,757 (21.8) | 30,527 (21.5) | 32,262 (21.9) | 32,170 (21.3) | 31,685 (22.0) | 33,662 (26.8) |
| Urogenital |  |  |  |  |  |  |  |  |  |  |  |
| Whole population, n (%) | 2,549 (5.3) | 3,921 (5.2) | 4,272 (5.0) | 5,459 (5.7) | 8,343 (6.6) | 9,456 (6.8) | 11,688 (7.3) | 12,363 (7.5) | 14,014 (8.4) | 14,644 (9.2) | 16,134 (11.4) |
| Shock group, n (%) | 172 (2.6) | 306 (2.8) | 308 (2.3) | 349 (2.7) | 430 (2.8) | 448 (2.8) | 523 (3.0) | 503 (2.9) | 566 (3.4) | 609 (3.9) | 613 (4.0) |
| Non-shock group, n (%) | 2,377 (5.8) | 3,615 (5.6) | 3,964 (5.5) | 5,110 (6.2) | 7,913 (7.1) | 9,008 (7.3) | 11,165 (7.9) | 11,860 (8.0) | 13,448 (8.9) | 14,035 (9.7) | 15,521 (12.3) |
| Bone and soft tissue |  |  |  |  |  |  |  |  |  |  |  |
| Whole population, n (%) | 2,189 (4.6) | 3,367 (4.4) | 4,195 (4.9) | 4,741 (5.0) | 6,629 (5.2) | 7,355 (5.3) | 8,630 (5.4) | 9,155 (5.6) | 9,436 (5.6) | 8,914 (5.6) | 9,331 (6.6) |
| Shock group, n (%) | 298 (4.5) | 508 (4.6) | 652 (4.9) | 721 (5.5) | 860 (5.6) | 976 (6.1) | 1,064 (6.2) | 1,132 (6.6) | 1,155 (6.8) | 965 (6.1) | 1,111 (7.3) |
| Non-shock group, n (%) | 1,891 (4.6) | 2,859 (4.4) | 3,543 (4.9) | 4,020 (4.9) | 5,769 (5.2) | 6,379 (5.2) | 7,566 (5.3) | 8,023 (5.4) | 8,281 (5.5) | 7,949 (5.5) | 8,220 (6.5) |
| Blood |  |  |  |  |  |  |  |  |  |  |  |
| Whole population, n (%) | 42 (0.1) | 75 (0.1) | 118 (0.1) | 144 (0.2) | 163 (0.1) | 161 (0.1) | 201 (0.1) | 188 (0.1) | 154 (0.1) | 142 (0.1) | 146 (0.1) |
| Shock group, n (%) | 4 (0.1) | 12 (0.1) | 18 (0.1) | 19 (0.1) | 31 (0.2) | 21 (0.1) | 30 (0.2) | 23 (0.1) | 20 (0.1) | 24 (0.2) | 22 (0.1) |
| Non-shock group, n (%) | 38 (0.1) | 63 (0.1) | 100 (0.1) | 125 (0.2) | 132 (0.1) | 140 (0.1) | 171 (0.1) | 165 (0.1) | 134 (0.1) | 118 (0.1) | 124 (0.1) |
| Others |  |  |  |  |  |  |  |  |  |  |  |
| Whole population, n (%) | 7,519 (15.7) | 11,757 (15.5) | 13,212 (15.5) | 13,743 (14.4) | 15,408 (12.2) | 16,199 (11.7) | 16,934 (10.6) | 16,921 (10.3) | 15,100 (9.0) | 14,166 (8.9) | 14,981 (10.6) |
| Shock group, n (%) | 1,697 (25.8) | 2,855 (25.8) | 3,282 (24.6) | 2,940 (22.3) | 2,972 (19.2) | 2,883 (18.1) | 2,957 (17.2) | 2,819 (16.4) | 2,741 (16.3) | 2,417 (15.3) | 2,208 (14.5) |
| Non-shock group, n (%) | 5,822 (14.1) | 8,902 (13.7) | 9,930 (13.9) | 10,803 (13.2) | 12,436 (11.2) | 13,316 (10.8) | 13,977 (9.8) | 14,102 (9.6) | 12,359 (8.2) | 11,749 (8.2) | 12,773 (10.2) |
| The hospital day of antibiotic initiation ^a^ | |  |  |  |  |  |  |  |  |  |  |
| Whole population | 3.0 (1.0-16.0) | 2.0 (1.0-15.0) | 2.0 (1.0-14.0) | 2.0 (1.0-13.0) | 1.0 (1.0-10.0) | 1.0 (1.0-9.0) | 1.0 (1.0-8.0) | 1.0 (1.0-8.0) | 1.0 (1.0-7.0) | 1.0 (1.0-6.0) | 1.0 (1.0-6.0) |
| Shock group | 7.0 (1.0-24.0) | 7.0 (1.0-23.0) | 7.0 (1.0-23.0) | 6.0 (1.0-21.0) | 5.0 (1.0-18.0) | 4.0 (1.0-17.0) | 4.0 (1.0-17.0) | 4.0 (1.0-16.0) | 3.0 (1.0-15.0) | 3.0 (1.0-14.0) | 3.0 (1.0-14.0) |
| Non-shock group | 2.0 (1.0-14.0) | 2.0 (1.0-13.0) | 2.0 (1.0-13.0) | 1.0 (1.0-11.0) | 1.0 (1.0-8.0) | 1.0 (1.0-8.0) | 1.0 (1.0-7.0) | 1.0 (1.0-6.0) | 1.0 (1.0-6.0) | 1.0 (1.0-5.0) | 1.0 (1.0-6.0) |
| Mechanical ventilation |  |  |  |  |  |  |  |  |  |  |  |
| Whole population, n (%) | 32,283 (19.7) | 50,612 (19.8) | 60,961 (20.5) | 63,866 (19.5) | 75,932 (18.1) | 80,896 (17.7) | 86,743 (17.2) | 87,393 (16.8) | 85,413 (16.4) | 81,185 (16.6) | 77,135 (16.2) |
| Shock group, n (%) | 14,509 (52.1) | 23,644 (52.2) | 29,268 (51.9) | 28,994 (51.7) | 32,369 (50.0) | 33,539 (50.1) | 35,868 (49.6) | 35,006 (49.8) | 32,933 (49.1) | 30,648 (50.3) | 30,302 (49.6) |
| Non-shock group, n (%) | 17,774 (13.1) | 26,968 (12.8) | 31,693 (13.2) | 34,872 (12.9) | 43,563 (12.3) | 47,357 (12.2) | 50,875 (11.7) | 52,387 (11.7) | 52,480 (11.6) | 50,537 (11.8) | 46,833 (11.3) |
| Renal replacement therapy |  |  |  |  |  |  |  |  |  |  |  |
| Whole population, n (%) | 10,924 (6.7) | 16,982 (6.6) | 20,588 (6.9) | 21,242 (6.5) | 25,432 (6.1) | 26,816 (5.9) | 28,646 (5.7) | 29,010 (5.6) | 34,429 (6.6) | 32,736 (6.7) | 32,785 (6.9) |
| Shock group, n (%) | 5,100 (18.3) | 8,161 (18.0) | 10,194 (18.1) | 10,226 (18.2) | 11,522 (17.8) | 11,935 (17.8) | 12,728 (17.6) | 12,508 (17.8) | 12,650 (18.8) | 11,905 (19.5) | 11,872 (19.4) |
| Non-shock group, n (%) | 5,824 (4.3) | 8,821 (4.2) | 10,394 (4.3) | 11,016 (4.1) | 13,910 (3.9) | 14,881 (3.8) | 15,918 (3.7) | 16,502 (3.7) | 21,779 (4.8) | 20,831 (4.9) | 20,913 (5.1) |
| Hydrocortisone use |  |  |  |  |  |  |  |  |  |  |  |
| Whole population, n (%) | 11,315 (6.9) | 17,067 (6.7) | 21,932 (7.4) | 22,981 (7.0) | 25,203 (6.0) | 26,477 (5.8) | 28,126 (5.6) | 35,533 (6.9) | 41,454 (8.0) | 33,851 (6.9) | 31,864 (6.7) |
| Shock group, n (%) | 2,883 (10.3) | 4,773 (10.5) | 6,543 (11.6) | 6,431 (11.5) | 6,916 (10.7) | 7,240 (10.8) | 7,869 (10.9) | 10,000 (14.2) | 11,793 (17.6) | 10,600 (17.4) | 10,515 (17.2) |
| Non-shock group, n (%) | 8,432 (6.2) | 12,294 (5.8) | 15,389 (6.4) | 16,550 (6.1) | 18,287 (5.2) | 19,237 (4.9) | 20,257 (4.7) | 25,533 (5.7) | 29,661 (6.5) | 23,251 (5.4) | 21,349 (5.2) |
| ICU admission |  |  |  |  |  |  |  |  |  |  |  |
| Whole population, n (%) | 38,194 (23.4) | 60,670 (23.7) | 75,940 (25.6) | 84,411 (25.8) | 103,629 (24.8) | 111,143 (24.3) | 117,823 (23.3) | 120,557 (23.2) | 118,640 (22.8) | 113,743 (23.3) | 109,042 (23.0) |
| Shock group, n (%) | 13,471 (48.4) | 21,965 (48.5) | 28,155 (50.0) | 28,816 (51.4) | 33,380 (51.5) | 34,361 (51.3) | 36,454 (50.4) | 35,458 (50.4) | 33,924 (50.5) | 31,768 (52.2) | 31,253 (51.1) |
| Non-shock group, n (%) | 24,723 (18.2) | 38,705 (18.4) | 47,785 (19.9) | 55,595 (20.5) | 70,249 (19.9) | 76,782 (19.7) | 81,369 (18.8) | 85,099 (19.0) | 84,716 (18.7) | 81,975 (19.2) | 77,789 (18.8) |

BMI, body mass index; ICU, intensive care unit, ^a^ The data shown represent the median value along with the interquartile range.

**Temporal trends were evaluated using linear regression for continuous variables, and the Cochran–Armitage trend test for binary categorical variables.**

- **Linear regression results**are presented as estimated annual change (slope), model fit (R²), and P value:
  – Age – Whole population: slope = +0.345, *R²* = 0.93, *P* < 0.001
  – Age – Shock group: slope = +0.182, *R²* = 0.59, *P* = 0.0059
  – Age – Non-shock group: slope = +0.318, *R²* = 0.89, *P* < 0.001
  – The hospital day of antibiotic initiation – Whole population: slope = –0.173, *R²* = 0.69, *P* = 0.0014
  – The hospital day of antibiotic initiation – Shock group: slope = –0.482, *R²* = 0.92, *P* < 0.001
  – (Additional linear regression results for proportions are shown in Supplementary Table S7b.)
- **Cochran–Armitage trend test results** are shown as *Z* statistic and *P* value for binary variables:
  – Community-onset sepsis – Whole population: *Z* = 13.66, *P* < 0.001
  – Community-onset sepsis – Shock group: *Z* = 23.00, *P* < 0.001
  – Community-onset sepsis – Non-shock group: *Z* = 12.85, *P* < 0.001
  – Chronic respiratory disease – Whole population: *Z* = 4.13, *P* < 0.001
  – Chronic respiratory disease – Shock group: *Z* = 3.97, *P* < 0.001
  – Chronic respiratory disease – Non-shock group: *Z* = 4.14, *P* < 0.001
  – Chronic renal failure – Whole population: *Z* = 3.23, *P* = 0.0012
  – Chronic renal failure – Shock group: *Z* = 3.41, *P* < 0.001
  – (Additional Cochran–Armitage results for other binary variables are shown in Supplementary Table S7b.)

1. Trend analysis

| **Category** | **slope** | ***R²*** | ***Z*** | ***P*** |
| --- | --- | --- | --- | --- |
| Age |  |  |  |  |
| Whole population | +0.345 | 0.93 |  | < 0.001 |
| Shock group | +0.182 | 0.59 |  | 0.0059 |
| Non-shock group | +0.318 | 0.89 |  | < 0.001 |
| Sex (male) |  |  |  |  |
| Whole population |  |  | –6.67 | < 0.001 |
| Shock group |  |  | –8.45 | < 0.001 |
| Non-shock group |  |  | –6.16 | < 0.001 |
| BMI |  |  |  |  |
| Whole population | +0.017 | 0.78 |  | < 0.001 |
| Shock group | +0.038 | 0.86 |  | < 0.001 |
| Non-shock group | +0.021 | 0.85 |  | < 0.001 |
| Community-onset sepsis |  |  |  |  |
| Whole population |  |  | +13.66 | < 0.001 |
| Shock group |  |  | +23.00 | < 0.001 |
| Non-shock group |  |  | +12.85 | < 0.001 |
| Comorbidity |  |  |  |  |
| Hypertension |  |  |  |  |
| Whole population |  |  | +7.15 | < 0.001 |
| Shock group |  |  | +6.08 | < 0.001 |
| Non-shock group |  |  | +7.18 | < 0.001 |
| Diabetes mellitus |  |  |  |  |
| Whole population |  |  | +7.57 | < 0.001 |
| Shock group |  |  | +7.00 | < 0.001 |
| Non-shock group |  |  | +7.61 | < 0.001 |
| Malignant tumor |  |  |  |  |
| Whole population |  |  | –12.68 | < 0.001 |
| Shock group |  |  | –9.46 | < 0.001 |
| Non-shock group |  |  | –12.76 | < 0.001 |
| Ischemic heart disease |  |  |  |  |
| Whole population |  |  | +2.25 | 0.0245 |
| Shock group |  |  | +2.26 | 0.0239 |
| Non-shock group |  |  | +3.03 | 0.0024 |
| Heart failure |  |  |  |  |
| Whole population |  |  | +1.23 | 0.2196 |
| Shock group |  |  | +1.77 | 0.077 |
| Non-shock group |  |  | +1.48 | 0.1393 |
| Chronic respiratory disease | |  |  |  |
| Whole population |  |  | +4.13 | < 0.001 |
| Shock group |  |  | +3.97 | < 0.001 |
| Non-shock group |  |  | +4.14 | < 0.001 |
| Cerebrovascular disease |  |  |  |  |
| Whole population |  |  | –2.63 | 0.0086 |
| Shock group |  |  | –0.75 | 0.4505 |
| Non-shock group |  |  | –2.65 | 0.0079 |
| Chronic renal failure |  |  |  |  |
| Whole population |  |  | +3.23 | 0.0012 |
| Shock group |  |  | +3.41 | < 0.001 |
| Non-shock group |  |  | +3.33 | < 0.001 |
| Focus of infection |  |  |  |  |
| Respiratory |  |  |  |  |
| Whole population |  |  | –1.73 | 0.0836 |
| Shock group |  |  | –6.95 | < 0.001 |
| Non-shock group |  |  | –1.95 | 0.0508 |
| Abdominal |  |  |  |  |
| Whole population |  |  | +4.01 | < 0.001 |
| Shock group |  |  | +10.34 | < 0.001 |
| Non-shock group |  |  | +3.63 | < 0.001 |
| Urogenital |  |  |  |  |
| Whole population |  |  | +8.21 | < 0.001 |
| Shock group |  |  | +5.2 | < 0.001 |
| Non-shock group |  |  | +7.62 | < 0.001 |
| Bone and soft tissue |  |  |  |  |
| Whole population |  |  | +7.52 | < 0.001 |
| Shock group |  |  | +8.59 | < 0.001 |
| Non-shock group |  |  | +6.6 | < 0.001 |
| Blood |  |  |  |  |
| Whole population |  |  | –0.61 | 0.5403 |
| Shock group |  |  | +0.8 | 0.4227 |
| Non-shock group |  |  | –0.61 | 0.5403 |
| Others |  |  |  |  |
| Whole population |  |  | –7.73 | < 0.001 |
| Shock group |  |  | –11.4 | < 0.001 |
| Non-shock group |  |  | –6.57 | < 0.001 |
| \| The hospital day of antibiotic initiation \|  \|  \|  \| \| --- \| --- \| --- \| --- \| | |  |  |  |
| Whole population | –0.173 | 0.69 |  | 0.0014 |
| Shock group | –0.482 | 0.92 |  | < 0.001 |
| Non-shock group | –0.109 | 0.6 |  | 0.0051 |
| Mechanical ventilation |  |  |  |  |
| Whole population |  |  | –8.35 | < 0.001 |
| Shock group |  |  | –4.95 | < 0.001 |
| Non-shock group |  |  | –8.01 | < 0.001 |
| Renal replacement therapy |  |  |  |  |
| Whole population |  |  | –0.33 | 0.7436 |
| Shock group |  |  | +2.14 | 0.0324 |
| Non-shock group |  |  | +1.51 | 0.1321 |
| Hydrocortisone use |  |  |  |  |
| Whole population |  |  | +0.13 | 0.897 |
| Shock group |  |  | +4.89 | < 0.001 |
| Non-shock group |  |  | –1.21 | 0.2259 |
| ICU admission |  |  |  |  |
| Whole population |  |  | –1.91 | 0.0555 |
| Shock group |  |  | +2.72 | 0.0066 |
| Non-shock group |  |  | –0.19 | 0.8501 |

BMI, body mass index; ICU, intensive care unit
