# Supplementary material for: Epidemiology and outcomes of septic shock in Japan: a nationwide retrospective cohort study from a medical claims database by the Japan Sepsis Alliance (JaSA) study group
Source: Crit Care. 2025 Jul 16;29:309. doi: 10.1186/s13054-025-05556-8 (PMC12269265; doi:10.1186/s13054-025-05556-8)
Supplement: Supplementary file 2 — Additional file 2: Figure S1. Annual changes in sepsis cases and deaths in shock and non-shock sepsis. This figure presents annual data from 2010 to 2020. The number of patients with sepsis significantly increased in both the shock group (slope = +4,180.55 patients/year, R² = 0.69, P= 0.0018) and the non-shock group (slope = +24,439.73 patients/year, R²= 0.81, P = 0.0002). The number of deaths significantly increased in the non-shock group (slope =+3,224.08 deaths/year, R² = 0.73, P = 0.0008), while the trend in the shock group remained relatively stable (slope = +329.01 deaths/year, R² = 0.16, P = 0.2288). Marker shapes indicate the data type: circles denote the number of patients, and triangles indicate the number of deaths. Gray lines indicate patients with the non-shock group, and black lines indicate patients in the shock group. Error bars indicate 95% confidence intervals. [file 13054_2025_5556_MOESM2_ESM.pdf]

Figure S1. Annual changes in sepsis cases and deaths in shock and non-shock sepsis.

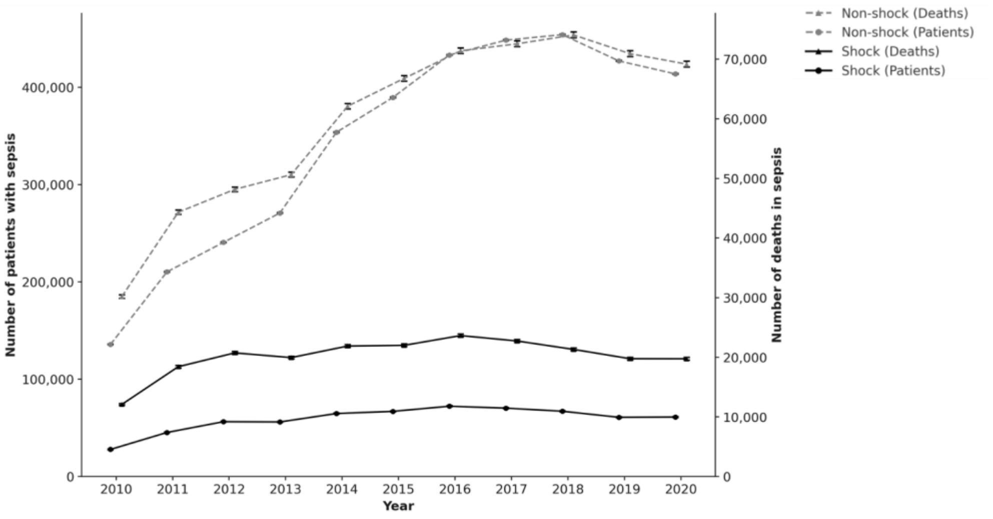

This figure presents annual data from 2010 to 2020. The number of patients with sepsis significantly increased in both the shock group (slope = +4,180.55 patients/year, $R^2 = 0.69$, $P= 0.0018$) and the non-shock group (slope = +24,439.73 patients/year, $R^2 = 0.81$, $P = 0.0002$). The number of deaths significantly increased in the non-shock group (slope = +3,224.08 deaths/year, $R^2 = 0.73$, $P = 0.0008$), while the trend in the shock group remained relatively stable (slope = +329.01 deaths/year, $R^2 = 0.16$, $P = 0.2288$). Marker shapes indicate the data type: circles denote the number of patients, and triangles indicate the number of deaths. Gray lines indicate patients with the non-shock group, and black lines indicate patients in the shock group. Error bars indicate 95% confidence intervals.
