# Supplementary material for: Epidemiology and outcomes of septic shock in Japan: a nationwide retrospective cohort study from a medical claims database by the Japan Sepsis Alliance (JaSA) study group
Source: Crit Care. 2025 Jul 16;29:309. doi: 10.1186/s13054-025-05556-8 (PMC12269265; doi:10.1186/s13054-025-05556-8)
Supplement: Supplementary file 3 — Additional file 3: Figure S2a.Annual trends in in-hospital mortality by age group in shock and non-shock sepsis. This figure presents annual data from 2010 to 2020. In-hospital mortality rates are shown by age group stratified in 10-year intervals (≤29, 30–39, 40–49, 50–59, 60–69, 70–79, and ≥80 years) among patients with septic shock and non-shock sepsis. Among patients with septic shock, all age groups, except for those aged ≤29 years, showed a significant decreasing trend in in-hospital mortality, with the strongest trend seen in patients aged ≥80 years (Z =–34.848, P < 0.0001). In non-shock patients with sepsis, all age groups exhibited significant downward trends (P < 0.0001 for all). Error bars indicate 95% confidence intervals. Statistical trends for each subgroup: Shock group≤29: Z=–1.897, P = 0.0578 30–39: Z=–3.614, P = 0.0003 40–49: Z=–6.882, P < 0.0001 50–59: Z=–12.827, P < 0.0001 60–69: Z=–20.937, P < 0.0001 70–79: Z=–30.125, P < 0.0001≥80: Z=–34.848, P < 0.0001 Non-shock group≤29: Z=–8.217, P < 0.0001 30–39: Z=–10.584, P < 0.0001 40–49: Z=–16.426, P < 0.0001 50–59: Z=–29.384, P < 0.0001 60–69: Z=–42.594, P < 0.0001 70–79: Z=–50.512, P < 0.0001≥80: Z=–54.085, P < 0.0001 Figure S2b.Annual changes in hospital stay by age group in shock and non-shock sepsis. This figure presents annual data from 2010 to 2020. The mean length of hospital stay (LOS, days) is shown by age group stratified in 10-year intervals (≤29, 30–39, 40–49, 50–59, 60–69, 70–79, and ≥80 years) among patients with septic shock and non-shock sepsis. In the shock group, a significant decrease in LOS was observed in patients aged 50–59 years and older, whereas there were no significant trends among younger age groups (≤49 years). In the non-shock group, LOS significantly decreased over time in all age groups, except for those aged ≤29 years (P = 0.3465). The steepest decline in LOS was observed in patients aged 30–39 years (slope =–1.99 days/year, R²= 0.57). Error bars indicate 95% confidence [file 13054_2025_5556_MOESM3_ESM.pdf]

Figure S2

a. Annual trends in in-hospital mortality by age group in shock and non-shock sepsis.

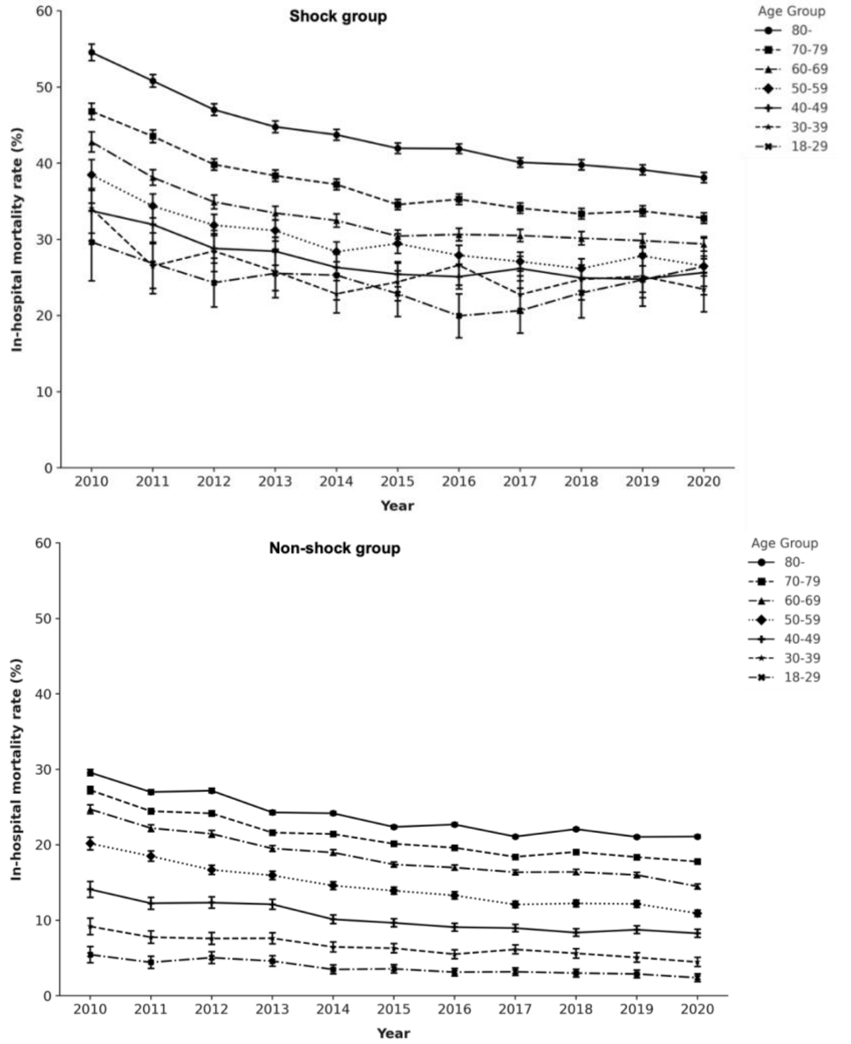

b. Annual changes in hospital stay by age group in shock and non-shock sepsis.

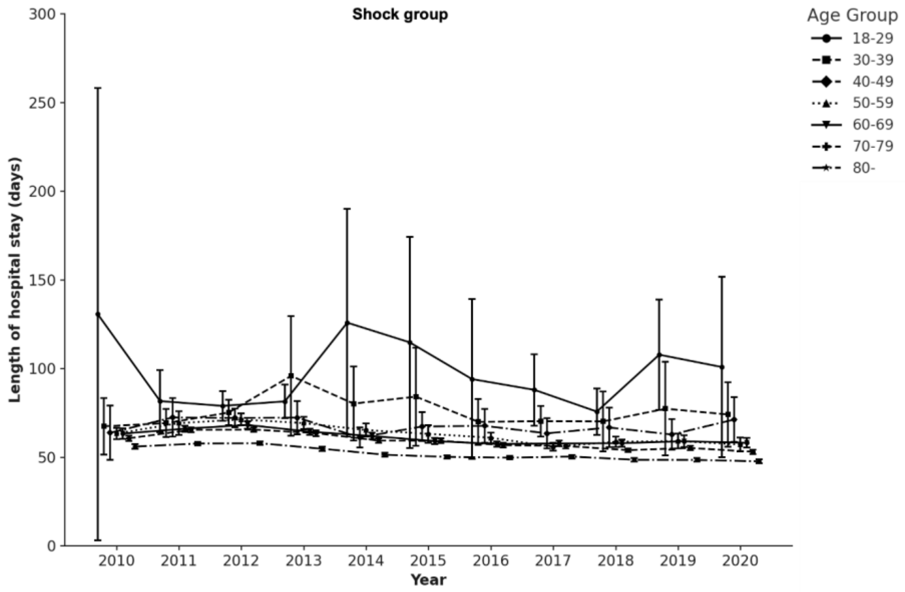

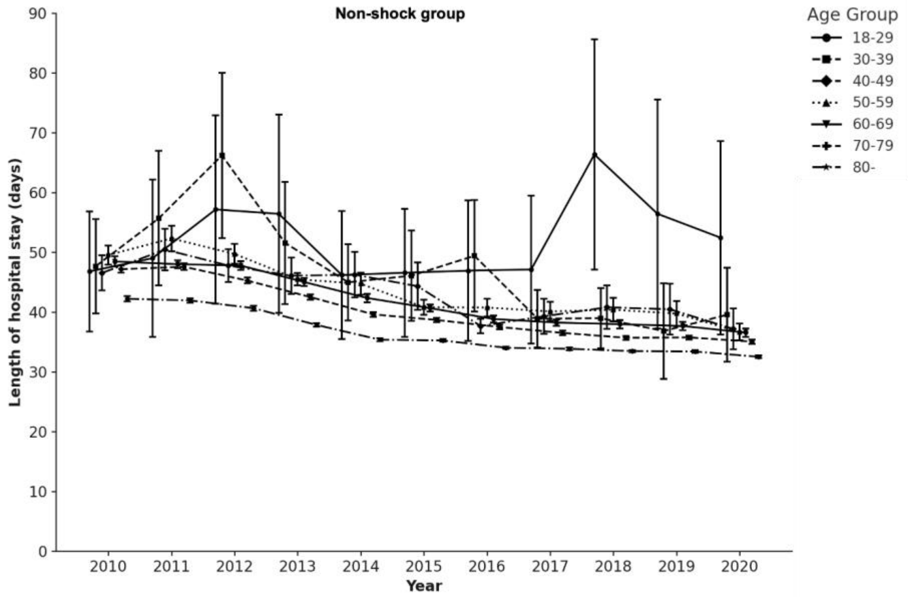

a. This figure presents annual data from 2010 to 2020. In-hospital mortality rates are shown by age group stratified in 10-year intervals (≤29, 30–39, 40–49, 50–59, 60–69, 70–79, and ≥80 years) among patients with septic shock and non-shock sepsis. Among patients with septic shock, all age groups, except for those aged ≤29 years, showed a significant decreasing trend in in-hospital mortality, with the strongest trend seen in patients aged ≥80 years ($Z = -34.848$, $P < 0.0001$). In non-shock patients with sepsis, all age groups exhibited significant downward trends ($P < 0.0001$ for all). Error bars indicate 95% confidence intervals.

Statistical trends for each subgroup:

Shock group

≤29: $Z = -1.897$, $P = 0.0578$

30–39: $Z = -3.614$, $P = 0.0003$

40–49: $Z = -6.882$, $P < 0.0001$

50–59: $Z = -12.827$, $P < 0.0001$

60–69: $Z = -20.937$, $P < 0.0001$

70–79: $Z = -30.125$, $P < 0.0001$

≥80: $Z = -34.848$, $P < 0.0001$

Non-shock group

≤29: $Z = -8.217$, $P < 0.0001$

30–39: $Z = -10.584$, $P < 0.0001$

40–49: $Z = -16.426$, $P < 0.0001$

50–59: $Z = -29.384$, $P < 0.0001$

60–69: $Z = -42.594$, $P < 0.0001$

70–79: $Z = -50.512$, $P < 0.0001$

≥80: $Z = -54.085$, $P < 0.0001$

b. This figure presents annual data from 2010 to 2020. The mean length of hospital stay (LOS, days) is shown by age group stratified in 10-year intervals (≤29, 30–39, 40–49, 50–59, 60–69, 70–79, and ≥80 years) among patients with septic shock and non-shock sepsis. In the shock group, a significant decrease in LOS was observed in patients aged 50–59 years and older, whereas there were no significant trends among younger age groups (≤49 years). In the non-shock group, LOS significantly decreased over time in all age groups, except for those aged ≤29 years ($P = 0.3465$). The steepest decline in LOS was observed in patients aged 30–39 years (slope = –1.99 days/year, $R^2 = 0.57$). Error bars indicate 95% confidence intervals.

Statistical trends for each subgroup:

Shock group

≤29: slope = –0.66, $R^2 = 0.01$, $P = 0.7362$

30–39: slope = –0.10, $R^2 = 0.01$, $P = 0.9120$

40–49: slope = –0.26, $R^2 = 0.04$, $P = 0.5457$

50–59: slope = –1.28, $R^2 = 0.65$, $P = 0.0026$

60–69: slope = –0.94, $R^2 = 0.67$, $P = 0.0021$

70–79: slope = –1.19, $R^2 = 0.80$, $P = 0.0002$

≥80: slope = –1.06, $R^2 = 0.86$, $P < 0.0001$

Non-shock group

≤29: slope = 0.61, $R^2 = 0.10$, $P = 0.3465$

30–39: slope = –1.99, $R^2 = 0.57$, $P = 0.0074$

40–49: slope = –1.18, $R^2 = 0.77$, $P = 0.0004$

50–59: slope = –1.43, $R^2 = 0.88$, $P < 0.0001$

60–69: slope = –1.34, $R^2 = 0.94$, $P < 0.0001$

70–79: slope = –1.37, $R^2 = 0.92$, $P < 0.0001$

≥80: slope = –1.03, $R^2 = 0.89$, $P < 0.0001$
