# Supplementary material for: Epidemiology and outcomes of septic shock in Japan: a nationwide retrospective cohort study from a medical claims database by the Japan Sepsis Alliance (JaSA) study group
Source: Crit Care. 2025 Jul 16;29:309. doi: 10.1186/s13054-025-05556-8 (PMC12269265; doi:10.1186/s13054-025-05556-8)
Supplement: Supplementary file 4 — Additional file 4: Figure S3a. Annual changes in in-hospital mortality by sex in shock and non-shock sepsis. This figure presents annual data from 2010 to 2020. In-hospital mortality rates are shown by sex among patients with septic shock and non-shock sepsis. Male patients exhibited consistently higher in-hospital mortality rates than female patients in both groups throughout the study period. Although the mortality rates significantly declined in all subgroups, male patients in the septic shock group showed the highest mortality rate (from 47.7% in 2010 to 34.4% in 2020), followed by female patients in the same group (from 45.2% to 31.4%). Similar decreasing trends were observed in patients with non-shock sepsis (male: from 28.0% to 19.7%; female: from 23.2% to 15.8%). Black lines represent patients with septic shock, and gray lines represent those with non-shock sepsis. Male patients are denoted by solid lines with circles, and female patients by dashed lines with squares. Error bars indicate 95% confidence intervals. Figure S3b.Annual changes in hospital stay by sex in shock and non-shock sepsis. This figure presents annual data from 2010 to 2020.Mean hospital length of stay (LOS, days) is shown by sex among patients with septic shock and non-shock sepsis. While the LOS was longer in male patients than in female patients in both groups, all subgroups demonstrated significant annual reductions. LOS decreased from 60.7 to 52.8 days in male patients with septic shock (slope =–1.14 days/year, R² = 0.80, P = 0.0002), from 61.5 to 55.0 days in female patients with septic shock (slope =–1.06 days/year, R² = 0.83, P= 0.0001), from 45.8 to 34.3 days in male patients with non-shock sepsis (slope =–1.28 days/year, R² = 0.92, P < 0.0001), and from 45.5 to 34.5 days in female patients with non-shock sepsis (slope =–1.25 days/year, R² = 0.90, P < 0.0001). Black lines represent patients with septic shock, and gray lines represent those with non-shock sepsis. Male patients are [file 13054_2025_5556_MOESM4_ESM.pdf]

Figure S3

a. Annual changes in in-hospital mortality by sex in shock and non-shock sepsis.

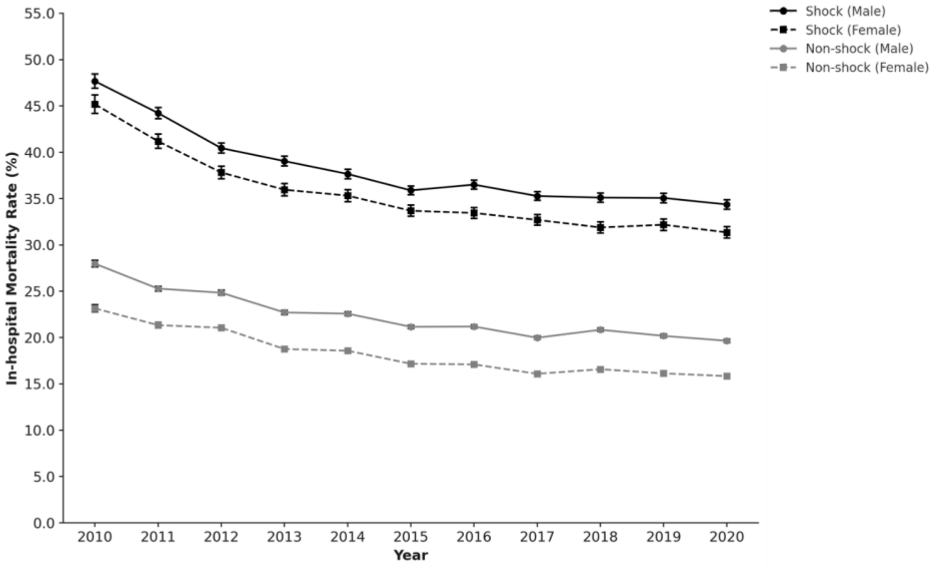

b. Annual changes in hospital stay by sex in shock and non-shock sepsis.

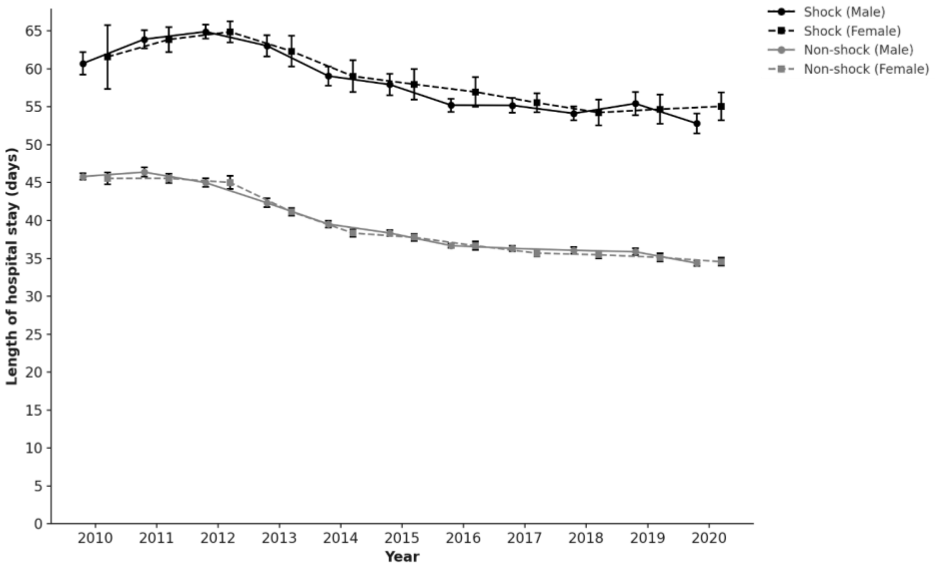

a. This figure presents annual data from 2010 to 2020. In-hospital mortality rates are shown by sex among patients with septic shock and non-shock sepsis. Male patients exhibited consistently higher in-hospital mortality rates than female patients in both groups throughout the study period. Although the mortality rates significantly declined in all subgroups, male patients in the septic shock group showed the highest mortality rate (from 47.7% in 2010 to 34.4% in 2020), followed

by female patients in the same group (from 45.2% to 31.4%). Similar decreasing trends were observed in patients with non-shock sepsis (male: from 28.0% to 19.7%; female: from 23.2% to 15.8%). Black lines represent patients with septic shock, and gray lines represent those with non-shock sepsis. Male patients are denoted by solid lines with circles, and female patients by dashed lines with squares. Error bars indicate 95% confidence intervals.

b. This figure presents annual data from 2010 to 2020. Mean hospital length of stay (LOS, days) is shown by sex among patients with septic shock and non-shock sepsis. While the LOS was longer in male patients than in female patients in both groups, all subgroups demonstrated significant annual reductions. LOS decreased from 60.7 to 52.8 days in male patients with septic shock (slope = –1.14 days/year, $R^2 = 0.80$, $P = 0.0002$), from 61.5 to 55.0 days in female patients with septic shock (slope = –1.06 days/year, $R^2 = 0.83$, $P = 0.0001$), from 45.8 to 34.3 days in male patients with non-shock sepsis (slope = –1.28 days/year, $R^2 = 0.92$, $P < 0.0001$), and from 45.5 to 34.5 days in female patients with non-shock sepsis (slope = –1.25 days/year, $R^2 = 0.90$, $P < 0.0001$). Black lines represent patients with septic shock, and gray lines represent those with non-shock sepsis. Male patients are represented by solid lines with circles, and female patients by dashed lines with squares. Error bars indicate 95% confidence intervals.
