# Supplementary material for: Epidemiology and outcomes of septic shock in Japan: a nationwide retrospective cohort study from a medical claims database by the Japan Sepsis Alliance (JaSA) study group
Source: Crit Care. 2025 Jul 16;29:309. doi: 10.1186/s13054-025-05556-8 (PMC12269265; doi:10.1186/s13054-025-05556-8)
Supplement: Supplementary file 5 — Additional file 5: Figure S4.Annual changes in in-hospital mortality by ICU admission and shock status. This figure presents annual data from 2010 to 2020. In-hospital mortality rates are shown across four subgroups based on ICU admission and shock status: shock with ICU admission, shock without ICU admission, non-shock with ICU admission, and non-shock without ICU admission. Throughout most of the study period, patients with septic shock who were not admitted to the ICU exhibited the highest mortality rates (from 50.5% in 2010 to 33.1% in 2020), whereas non-shock patients admitted to the ICU consistently showed the lowest mortality (from 22.9% to 17.1%). The gap in mortality between ICU and non-ICU subgroups within the shock group narrowed over time, with no statistically significant difference observed in 2019 and 2020. Black lines represent patients with septic shock, and gray lines represent those with non-shock sepsis. ICU-admitted patients are denoted by solid lines with circles, and non-ICU patients by dashed lines with squares. Error bars indicate 95% confidence intervals [file 13054_2025_5556_MOESM5_ESM.pdf]

**Figure S4.** Annual changes in in-hospital mortality by ICU admission and shock status.

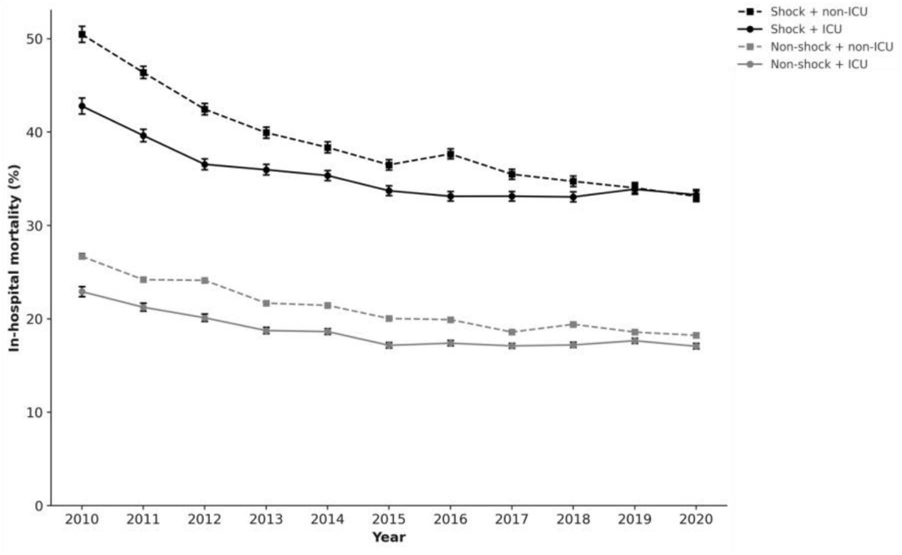


This figure presents annual data from 2010 to 2020. In-hospital mortality rates are shown across four subgroups based on ICU admission and shock status: shock with ICU admission, shock without ICU admission, non-shock with ICU admission, and non-shock without ICU admission. Throughout most of the study period, patients with septic shock who were not admitted to the ICU exhibited the highest mortality rates (from 50.5% in 2010 to 33.1% in 2020), whereas non-shock patients admitted to the ICU consistently showed the lowest mortality (from 22.9% to 17.1%). The gap in mortality between ICU and non-ICU subgroups within the shock group narrowed over time, with no statistically significant difference observed in 2019 and 2020. Black lines represent patients with septic shock, and gray lines represent those with non-shock sepsis. ICU-admitted patients are denoted by solid lines with circles, and non-ICU patients by dashed lines with squares. Error bars indicate 95% confidence intervals.
